# Supplementary material for: Protein-Peptide Turnover Profiling reveals the order of PTM addition and removal during protein maturation
Source: Nat Commun. 2022 Dec 2;13:7431. doi: 10.1038/s41467-022-35054-2 (PMC9718778; doi:10.1038/s41467-022-35054-2)
Supplement: Supplementary file 1 — Supplementary Information [file 41467_2022_35054_MOESM1_ESM.pdf]

Supplementary information

Protein-Peptide Turnover Profiling reveals the order of PTM addition and removal during protein maturation

Hammarén, Geissen *et al.*

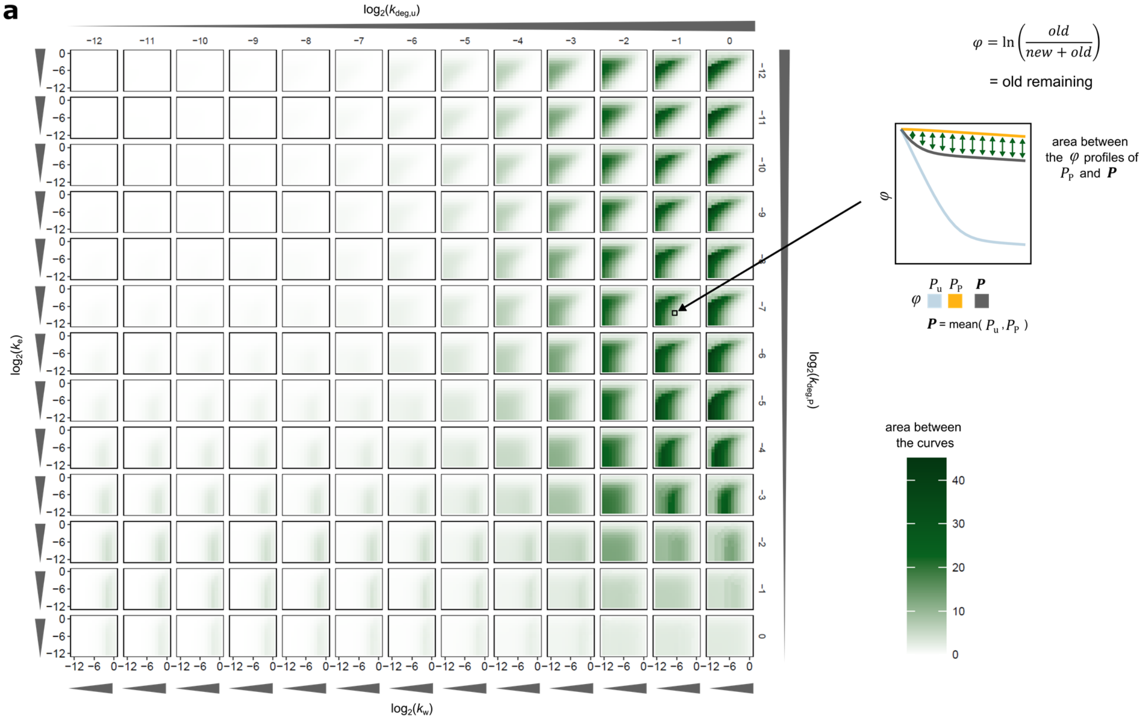

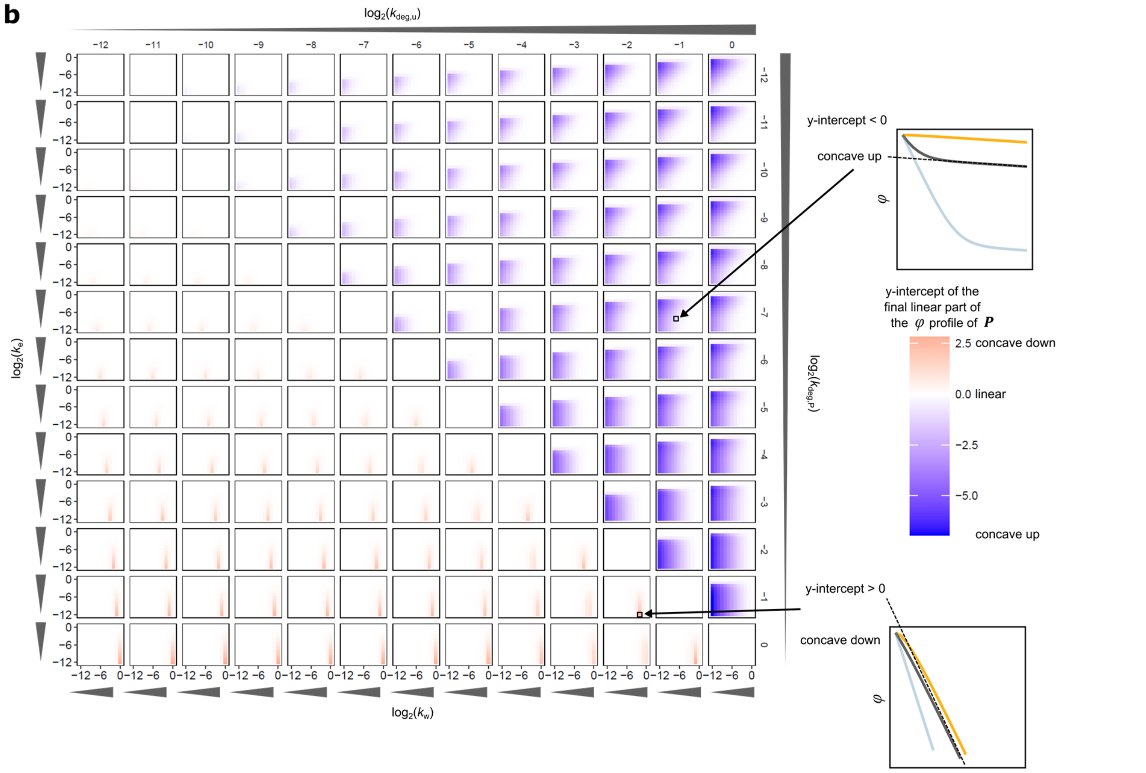

Supplementary Figure 1: Example output of Model 1:2 with different parameter combinations. a, Area between the clearance profile of the modified species, P_P, and the entire protein pool P for a simulation of 0-24 h varying parameters $k_{deg,u}$, $k_{deg,P}$, k_w, and k_e. Darker colors reflect larger differences in clearance profile. Note that the distance is always

positive, as the modified species always exhibits slower or similar clearance than the entire protein pool(Model 1:2). For calculating the difference, values of φ corresponding to less than ~0.3% old remaining were omitted as smaller ratios are not practically measurable with pSILAC-MS. **b**, Same parameter combinations as in (a), but showing the shape of the clearance profile of the entire protein pool *P* by plotting the extrapolated y-intercept of the linear end part of the curve. Nonlinear concave up curves yield a negative y-intercept (blue), while concave down curves give positive y-intercepts (red). Note that while the largest differences in clearance (a) can be achieved when $k_{deg,u}$ >> $k_{deg,P}$, this parameter combination simultaneously leads to a strongly nonlinear, concave up clearance curve (b) for the entire protein pool *P*. k_{syn} was not varied and is not shown, as it does not affect the clearance profiles (see Supplementary Note 1).

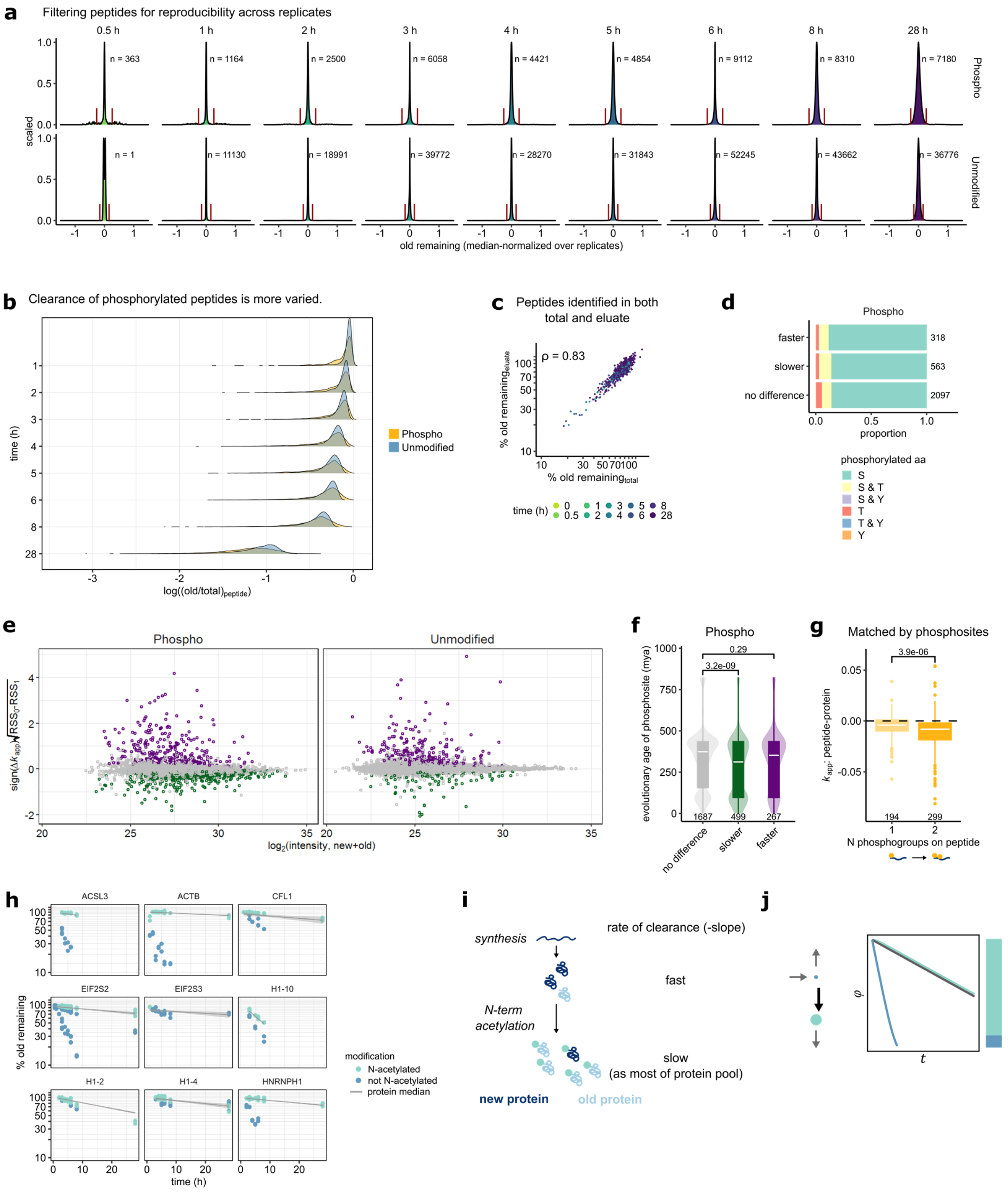

Supplementary Figure 2: Experimental measurement of clearance rates with PPToP. a, Filtering of clearance values (from SILAC ratios) for reproducibility across replicates. **b**, Phosphopeptides show more varied clearance than unmodified peptides. Distributions of raw clearance values (before correction for cell division) of peptides from proteins with at least a single identified phosphopeptide are shown (medians over replicates). **d,** High reproducibility of clearance measured for peptides quantified in both eluate and flow-through of the phosphoenrichment procedure shows that enrichment does not introduce artifacts in measured SILAC ratios. Shown are clearance values corrected for cell cycle and filtered for reproducibility across replicates. ρ, Spearman correlation. **d**, Hits from PPToP are evenly

distributed across different phosphorylated residues, as well (**e**) as across peptide abundance. **f**, Phosphopeptide hits with slower clearance are evolutionarily younger than the other measured phosphosites. Evolutionary age from Ref [1]. Two-sided Wilcoxon test. Numbers below indicate the number of unique modified peptides in each group. **g**, Addition of second phosphate group causes decrease in clearance rate. Related to Figure 3 e. Shown are peptides including exactly two detected phosphosites within their boundaries, and which are phosphorylated either once or twice. Peptides detected to have faster clearance were omitted as in Figure 3 e. Unpaired, two-sided *t*-test ($t = 4.67$, df = 490.64). Including peptides with significantly faster clearance does not change the direction, nor significance of the effect ($p = 3e-4$). **h**, Representative plots of proteins undergoing N-terminal acetylation (N-Ac). Non-acetylated N-terminal peptides are shown in blue and the corresponding acetylated forms in teal. The median clearance of the protein is shown as a gray line. **i**, Cartoon of the linear protein maturation cascade of irreversible protein N-Ac. **j**, Model 1:2 showing a theoretical clearance profile for the unmodified species (in blue), the modified species (in teal) as well as the entire protein pool (*P*, in gray) for rapid, irreversible protein modification, such as N-Ac. Boxplots in **f** and **g** consist of median line, box: upper and lower quartiles, whiskers: 1.5 times interquartile range, points: outliers. Source data are provided as a Source Data file.

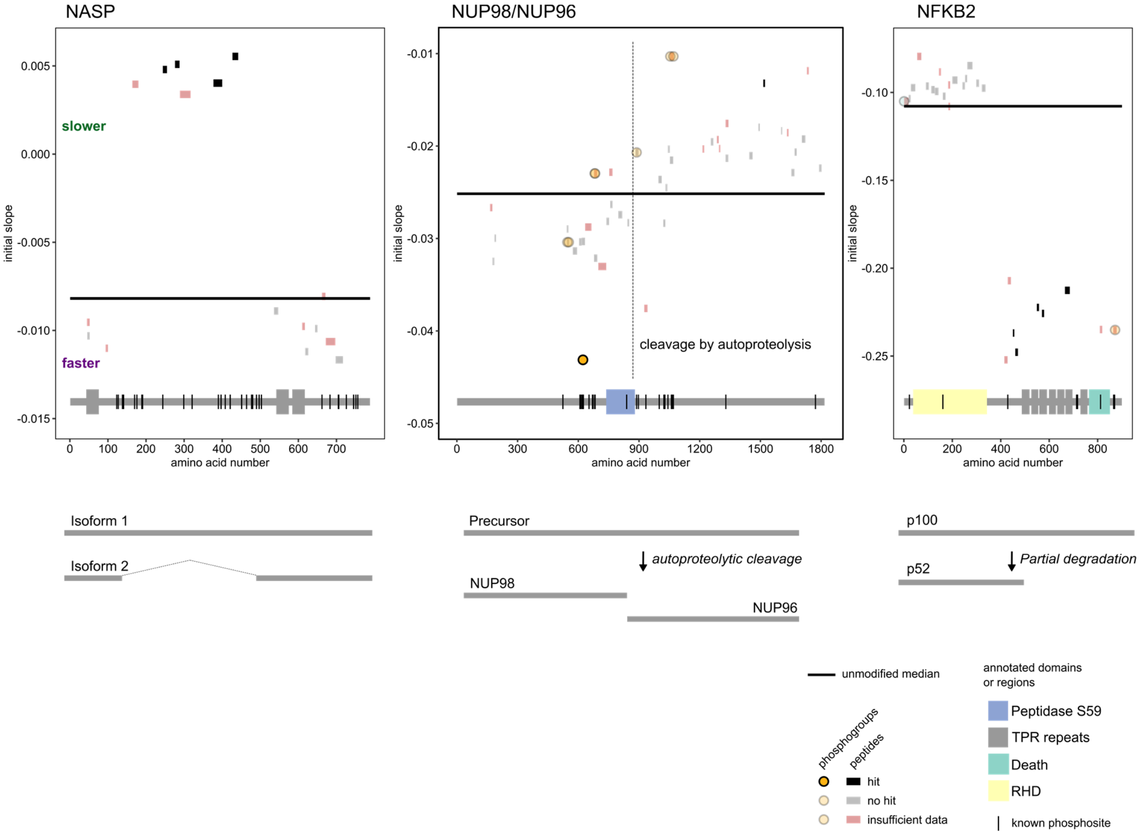

Supplementary Figure 3: Protein-level turnover effects uncovered by PPToP. Three examples of intra-protein differential clearance rates due to protein-level effects such as alternative splicing generating isoforms with differing turnover (Nuclear autoantigenic sperm protein, NASP, left panel), post-translational protein cleavage to generate two proteins from a single precursor (Nucleoporins 96 and 98, NUP96/98, middle panel), or partial protein degradation generating a signaling competent proteoform (Nuclear factor NF-kappa-B, NFKB2, right panel). Each line segment (black or gray) shown represents a unique modified peptide detected along the protein's sequence. The median initial clearance rate (k_{app}) over all replicates is shown on the y axis for each peptide. The median initial clearance rate fitted to all unmodified peptides is shown as a dark line. Hits from the comparative fitting approach are indicated. Detected phosphosites are shown with spheres. Underneath is a cartoon depiction of the protein's primary sequence with annotated domains and known phosphorylation sites (from Uniprot).

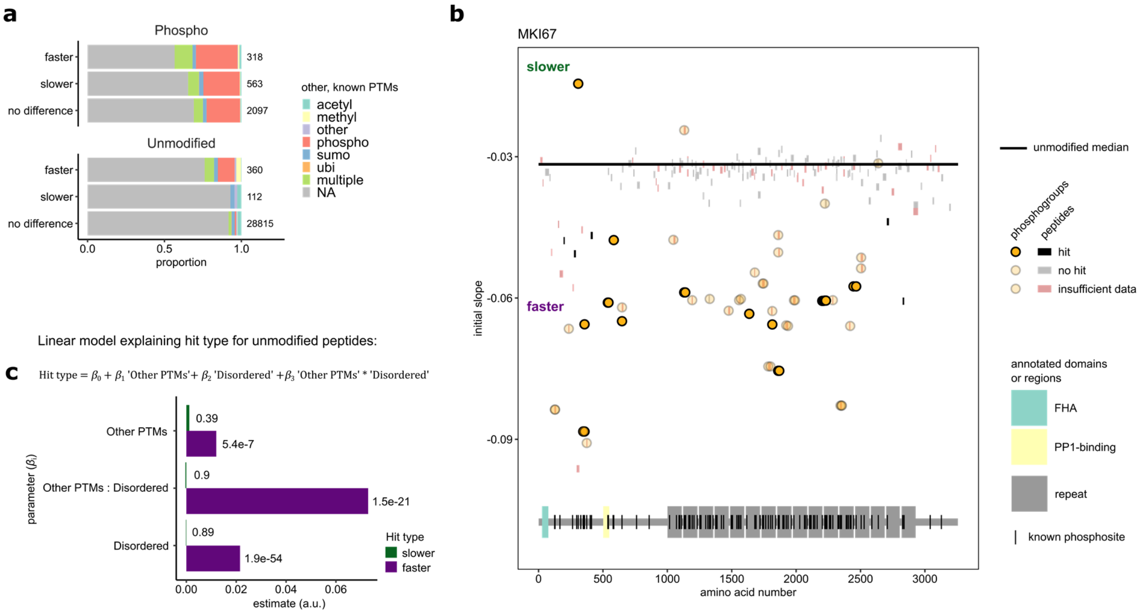

Supplementary Figure 4: Faster clearance due to other PTMs, concerted dephosphorylation, and enrichment of disordered regions in peptides with faster clearance.a, Distribution of other, known PTMs (from Uniprot) mapping to peptides in our PPToP dataset. **b**, Overview of PPToP data for MKI67 showing substantial number of phosphopeptides with similarly faster clearance. See https://apps.embl.de/pptop for an interactive web application to browse and visualize PPToP data for all proteins quantified. **c**, Peptides with faster clearance are enriched in disordered regions even after correcting for the enrichment of other, known PTMs. Linear regression model used to fit to distribution of slower/faster unmodified peptides. Shown are estimates for the parameters (β) and associated p values for the parameter being non-zero (i.e. likelihood that that the predictor variable does not predict the response variable (Hit type)). Source data are provided as a Source Data file.

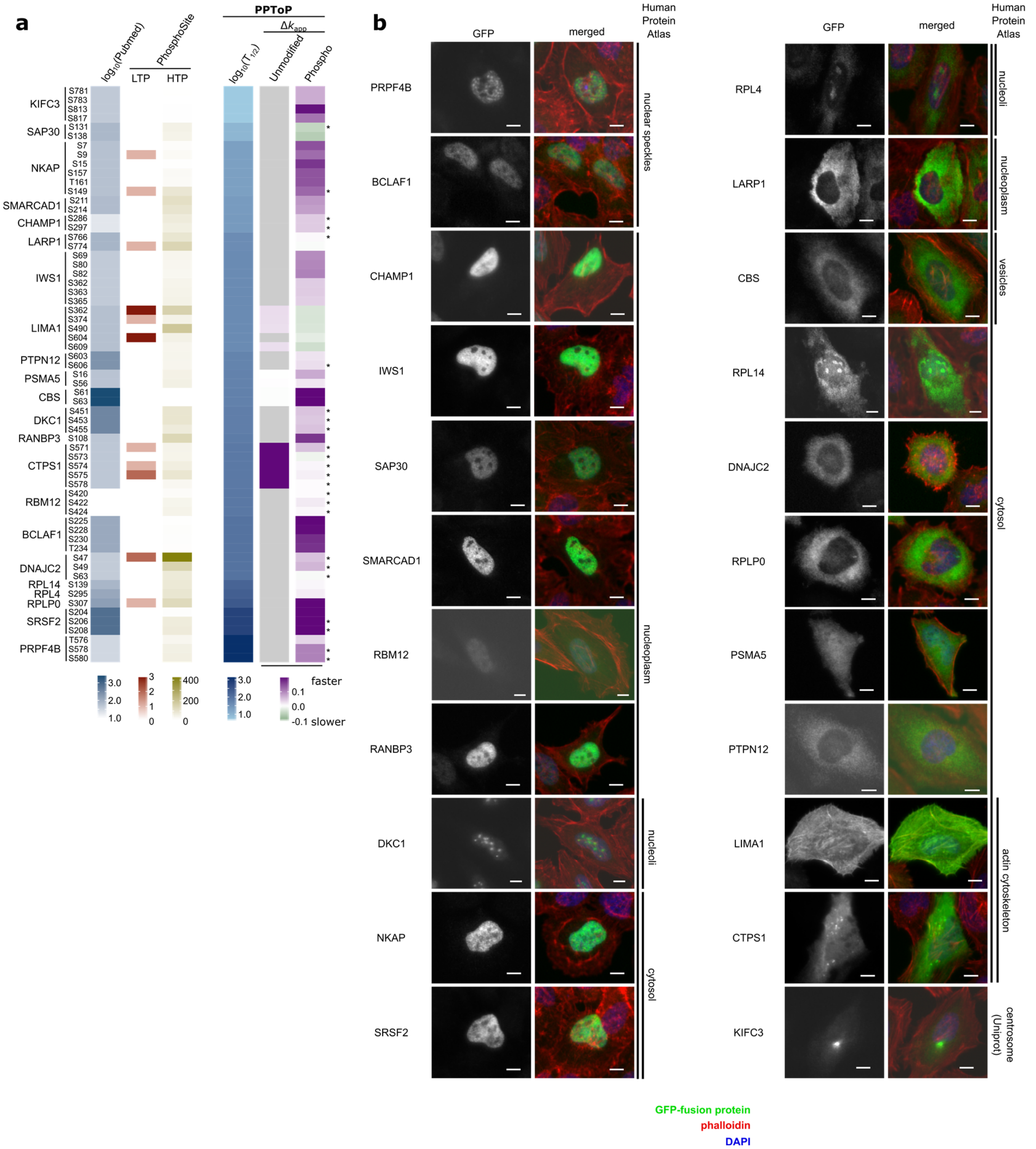

Supplementary Figure 5: GFP-fusion constructs of proteins of interest localize correctly upon exogenous expression. a, Target phosphosites chosen for follow-up experiments. Representative targets were chosen to include a diverse mix of previous information available in the literature, as well as covering a wide range of protein half-lives ($\log_2(T_{1/2})$ in hours, this study). Differential clearance of the unmodified or phosphorylated forms carrying the phosphosites of interest are shown. Cases, where the same phosphosite is present on multiple peptides with differing clearance rates are marked with asterisks. Cases where the unmodified form of the phosphosite was not quantified are shown in gray. LTP = Low-throughput, HTP = high-throughput reference studies. **b**, Representative micrographs of transiently-transfected HeLa cells stained for genomic DNA (DAPI in blue), filamentous actin (phalloidin in red), and the GFP-fusion protein (GFP in green). Annotations on the right are the primary subcellular locations given in the Human Protein Atlas (https://www.proteinatlas.org/). Note the multiple

annotations for several proteins. Scale bar = 10 μm. Figure shows representative micrograph from a single transfection of each WT construct. Source data are provided as a Source Data file.

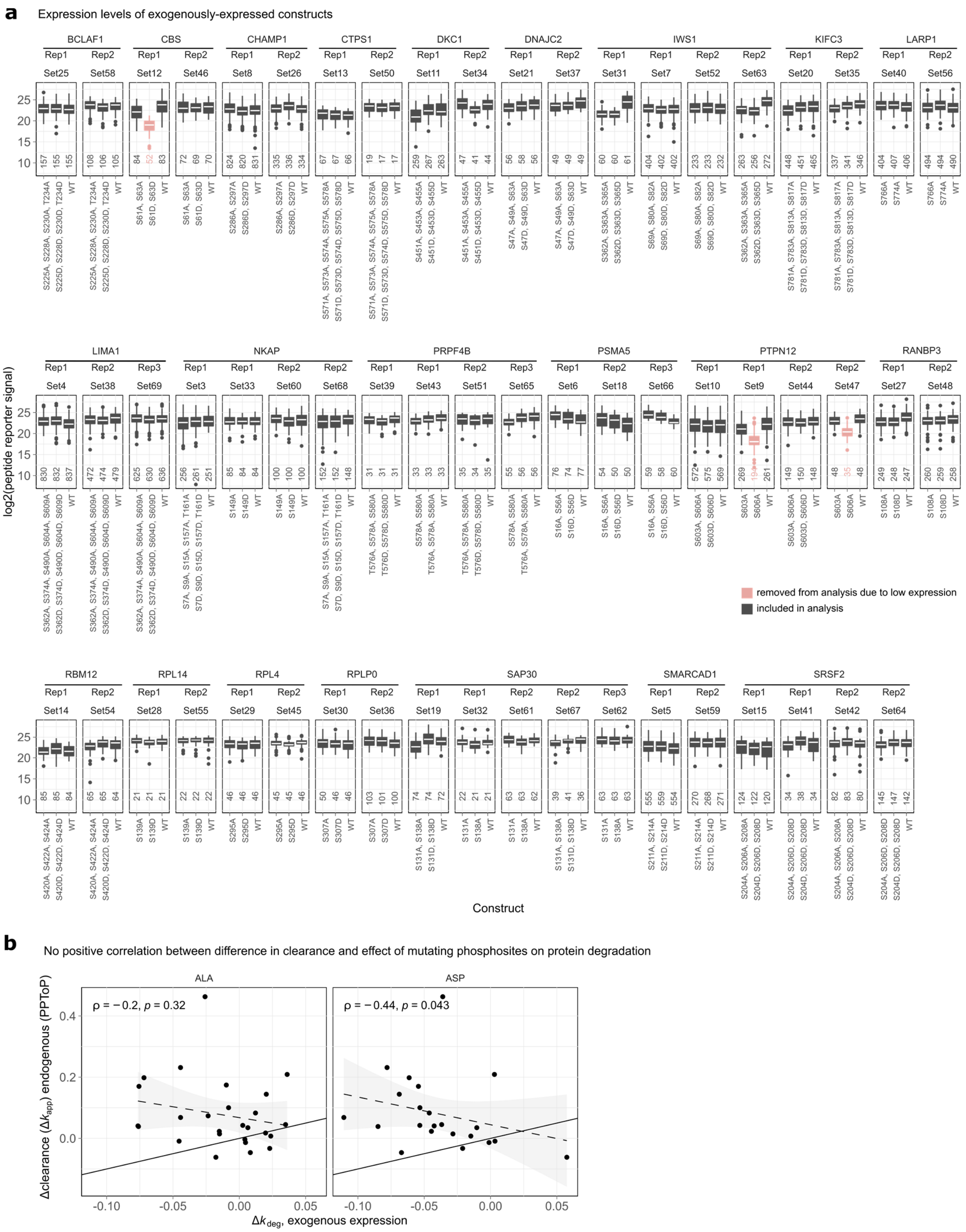

Supplementary Figure 6: Exogenous expression of GFP-fusion constructs. a, TMT reporter signals for light-labeled peptides of each construct at time-point 0 h of the SILAC time course (see Figure 5 a). Constructs with median expression >5-fold lower than the median of all constructs in that set were excluded from analysis (shown in pink). Each facet represents data from a single TMT experiment. Numbers below boxplots are individual peptide-spectrum matches with non-zero TMT reporter signal intensity of the protein in question. Boxplots consist of median line, box: upper and lower quartiles, whiskers: 1.5 times

interquartile range, points: outliers. **b**, No positive correlation between the difference in clearance rate of phosphorylated peptides compared to the protein median (Δk_{app}) and the difference in actual degradation constants between mutants of the same phosphorylation sites and the wild-type protein (Δk_{deg}). Spearman correlation. p value for null hypothesis testing, i.e. no correlation (two-sided). The shaded region indicates the 95% confidence interval of the fit. Source data are provided as a Source Data file.

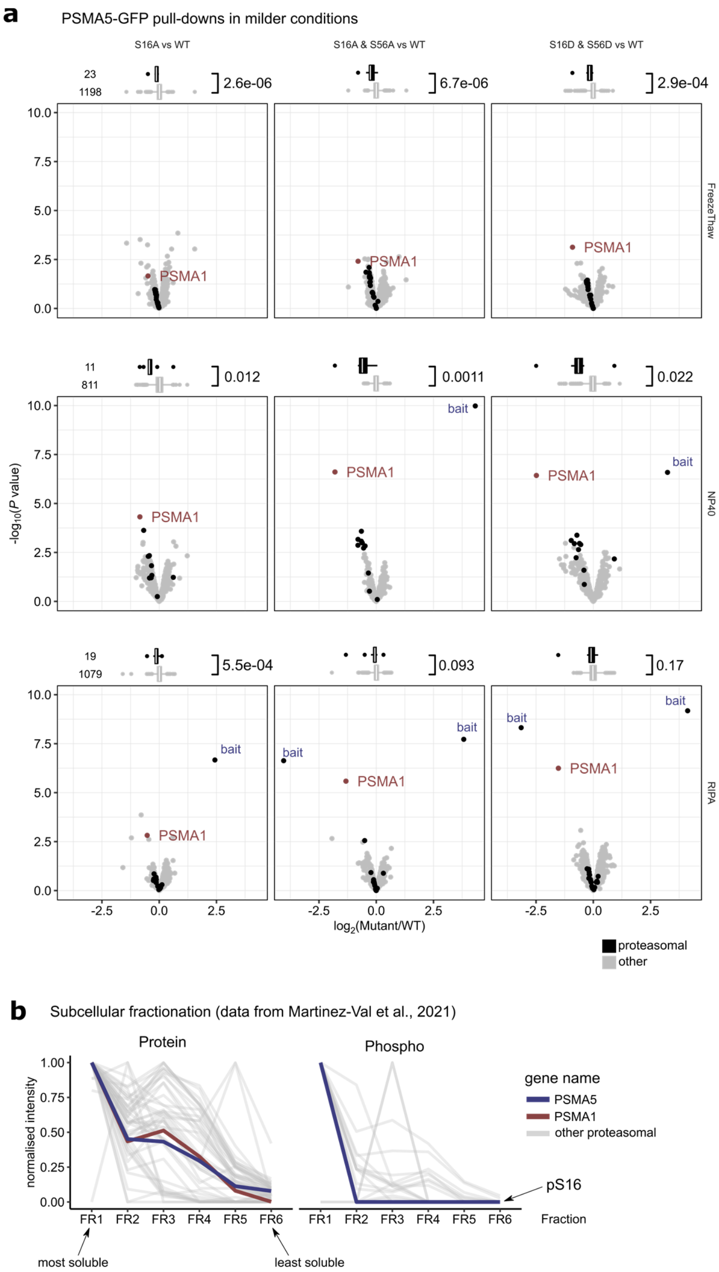

Supplementary Figure 7: PSMA5 S16 phosphorylation affects incorporation into proteasome. a, Pull-downs of exogenously expressed PSMA5-GFP constructs from HeLa cells in different buffer conditions. Milder buffer conditions (FreezeThaw and NP40) preserve protein-protein interactions and show reduced co-pulldown of proteasomal proteins with S16 mutant PSMA5-GFP constructs compared to wild-type PSMA5-GFP. x axis shows fold change of indicated mutant constructs over wild-type. y axis is the unadjusted *p* value from a limma analysis. N = 3 biological replicates. . Boxplots above show comparisons of proteasomal proteins to all other proteins identified in the pull-down excluding the bait proteins themselves: *p* values of two-tailed *t* tests. Numbers indicate number of proteins in each group. **b**, Subcellular fractionation of endogenous proteasomal proteins from unstimulated HeLa cells shows that S16 phosphorylated PSMA5 is only detected in the most soluble fraction (FR1)

likely to also include free proteasomal subunits, whereas PSMA5 (in blue), PSMA1 (in red), as well as other proteasomal proteins (in grey) are also present in fractions containing less soluble protein assemblies (FR2-FR6). Intensity normalised to the highest value for each protein. Shown are median values of four replicates. Data from Ref [2]. Source data are provided as a Source Data file.

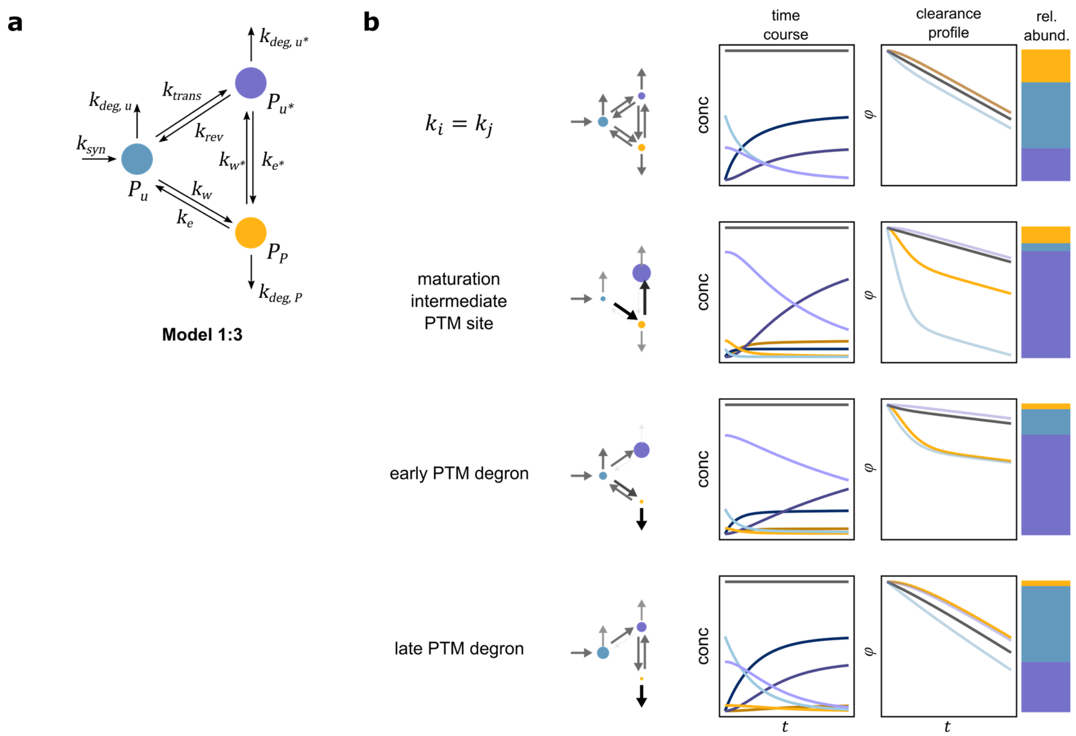

Supplementary Figure 8: A three-species protein turnover and modification model enables modeling phosphodegrons and intermediate species as species with faster clearance. a, Model structure of Model 1:3 with two distinct unmodified species (P_u and P_{u^*}), which could represent, for instance, young and old protein, or distinct protein pools defined by some other means, such as other PTMs, distinct subcellular localization, protein-protein interaction assemblies, etc. See Supplementary Note 1 for full description of the model. **b**, Example profiles allowed by Model 1:3. First row: all parameters are of the same magnitude. The "early degron" (third row) could, for instance, represent a quality control step early in a protein's lifetime, where the protein either is modified (and subsequently rapidly degraded), or matures into a more stable form (represented by P_{u^*}). Note that both a maturation intermediate modified pool (second row) as well as an early degron will lead to faster clearance of the modified species P_P. A "late degron" (bottom row) represents a protein, which is modified only at a later stage during its lifetime. Despite the modified species P_P having low proteolytic stability ($k_{deg,P}$ is high), the clearance profile for P_P is slower than for the entire protein pool P due to the large distance between synthesis and the modified form in the model.

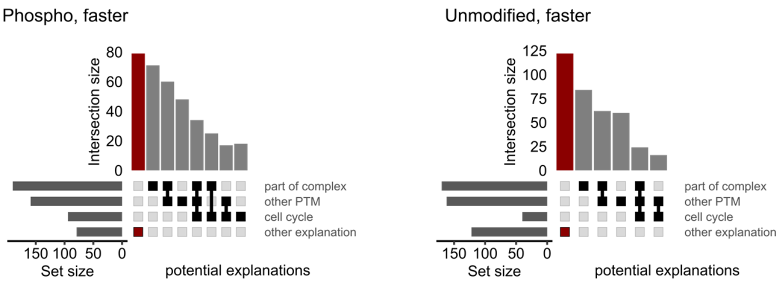

Supplementary Figure 9: Summary of potential explanations of peptides with faster clearance identified by PPToP. A significant proportion of hits lack a straightforward explanation based on previous data, thus representing an interesting starting point for further discovery. Explanations taken from published sources as follows: part of complex, CORUM[3]; other PTM, Uniprot[4,5]. Source data are provided as a Source Data file.

Supplementary Note 1 on
Protein-Peptide Turnover Profiling reveals the order of PTM addition and removal during protein maturation

Hammarén et Geissen, et al.

November 16, 2022

Contents

1 Supplementary Note 1 overview **2**

2 Introduction to the models **2**
2.1 Modelling assumptions 2
2.2 Terminology and notation 2

3 Model 0:1 - a single homogeneous protein pool **3**
3.1 M0:1 Model definition 3
3.2 M0:1 Clearance profile and clearance rate 4
3.3 M0:1 Summary 4
3.4 A single homogeneous protein pool - Correction for cell cycle related increase in protein abundance 4

4 Model 1:2 - synthesis followed by modification **5**
4.1 M1:2 Model definition 6
4.2 M1:2 Model reduction 6
4.3 M1:2 Analytical solutions for model species time course 8
4.4 M1:2 Clearance profile and clearance rate 8
4.5 M1:2 the PTM clearance rate is always less or equal to the clearance rate of the entire protein pool 9
4.6 M1:2 clearance profile curvature as measured by the y-axis intercept of the linear section of the profile 9
4.7 M1:2 Structural identifiability analysis 11
4.8 M1:2 Summary 12

5 Model 1:3 - alternative path to modification via an additional protein species **13**

6 General analysis of clearance profiles and clearance rates **14**

Supplementary References **15**

1 Supplementary Note 1 overview

This Supplementary Note contains the theoretical considerations regarding models encoding different wiring schemes of post-translational modifications (PTMs). We use these models to analyse how the clearance profile patterns of Protein-Peptide Turnover Profiling (PPToP) experiments depend on the assumed underlying wiring schemes.

Throughout this Supplementary Note, we describe and analyse the individual models one by one. The simplest case, where a given protein is modelled as a single, homogeneous pool, has been previously described [6, 7]. However, we will use it here to introduce the terminology and illustrate correction of the PPToP data for expansion of the protein pool by cell growth in Section 3.

All symbolic and numeric computations where performed in MATLAB [8]. The parameter sample for the plots for Supplementary Figures 10 to 13 in Section 4.6 was generated with the latin hypercube sampling algorithm [9] and Supplementary Figures 10 to 13 were generated with the hexscatter function from [10].

2 Introduction to the models

The following analysis is based on ordinary differential equations (ODEs) describing the dynamics of a metabolic labelling experiment (Figure 1 a). The ODEs are derived by modelling the wiring scheme based on biochemical reactions obeying mass action kinetics. While we focus mainly on proteoforms defined by the addition and removal of PTMs, the same considerations and conclusions apply to any analogous metabolic labeling experiment of biomolecules in which a species can exist in multiple measurable states that can interconvert, such as nucleic acids and their modifications [11].

2.1 Modelling assumptions

We built our ODE models on the following assumptions

1. Protein synthesis is zeroth order.
2. Degradation, as well as addition and removal of a modification are first order and random (i.e. independent of the molecular age of a protein molecule, meaning that the probability of a protein to participate in a reaction is the same for each protein molecule and constant).
3. The initial conditions correspond to the pre-label-switch steady state.
4. Label switch is near-instantaneous, complete, and label-equilibration times are negligible, as is usually the case in cell culture experiments, where medium exchange is near-total and efficient. Discussion on experimental setups involving slow label equilibration and resulting partial labelling has been provided elsewhere (see, e.g., [12–14]).
5. The label does not affect protein stability or phosphorylation/dephosphorylation kinetics.
6. The data come either from non-dividing cells (constant steady state protein abundance) or have already been corrected for cell cycle-related increase of the protein abundance during data collection. (See Section 3.4 on how to correct for cell cycle effects.)

2.2 Terminology and notation

In the models we refer to protein and peptide species by P. No subscript indicates the entire protein pool, and individual peptide species are indicated by subscripts, e.g. P_u and P_P for the unmodified and post-translationally modified species, respectively. To distinguish between entities that where synthesized before and after the label switch, we append 'old' and 'new' to the subscript, respectively. In the main text we do not distinguish between 'old' and 'new' in the notation for model species and in illustrations of the models. We do this for clearance of presentation and because it is possible to express the dynamics of our full models (new and old species) by the dynamics of the old peptide species only, as shown representatively for Model 1:2 in Section 4.2.

The fractional rate constants, that is which fraction of a peptide species is converted per unit time by a reaction, are noted by the letter k with a subscript indicating the respective conversion reaction.

These rate constants are the (unknown) parameters of a model and assumed to be positive. We use $\frac{\mathrm{d}}{\mathrm{d}t}$ to indicate the derivative of a quantity with respect to time.

The definition of a model comprises three components: the ODEs describing the species' dynamics, the initial conditions defining the amount of each species at the time of label switch, and the observables encoding the experimental setup by expressing the available data in terms of the model species and parameters. The model name 'Model x:y' encodes the number of individual PTM species 'x' and the total number of model species 'y'.

We distinguish between clearance profile and clearance rate to quantitatively describe the readout of PPToP experiments. With *clearance profile* we refer to the time course of the remaining fraction of protein that was synthesized pre-label switch (old protein). With *clearance rate* we refer to the slope of this time course which describes the speed at which the old protein disappears. Our *clearance rate* therefore corresponds to the *turnover rate* in traditional protein turnover experiments. However, we term it clearance rate to distinguish it from turnover, which is commonly associated with degradation, while we study combined effects of degradation and modification.

3 Model 0:1 - a single homogeneous protein pool

In the simplest case a protein is modeled as one homogeneous pool (Figure 1 c). We term this system Model 0:1 (short M0:1, for 0 individual PTM species and 1 protein species in total). In the following subsections we define Model 0:1 by means of its dynamics, initial conditions and observables, derive what we term the clearance profile and clearance rate and recapitulate how to correct for a cell cycle-caused increase in protein after the label switch.

3.1 M0:1 Model definition

Model 0:1 describes the dynamics after the label switch ($t = 0$) as:

$$\frac{\mathrm{d}}{\mathrm{d}t}\mathrm{P}_{old}(t) = -\mathrm{P}_{\mathrm{old}}(t)\, k_{deg} \tag{1}$$

$$\frac{\mathrm{d}}{\mathrm{d}t}\mathrm{P}_{new}(t) = -\mathrm{P}_{new}(t)\, k_{deg} + k_{syn} \tag{2}$$

with initial conditions

$$\mathrm{P}_{old}(0) = \mathrm{P}_{old,0} = \frac{k_{syn}}{k_{deg}} \tag{3}$$

$$\mathrm{P}_{new}(0) = 0, \tag{4}$$

where $\mathrm{P}_{old}(t)$ is the number of molecules of protein P that where synthesized prior to the label switch which are present at time t after the label switch, $\mathrm{P}_{new}(t)$ is the number of molecules of protein P that have been newly synthesized until time t after the label switch, k_{syn} is the synthesis rate, and k_{deg} the degradation constant of the protein.

The initial condition of the old species, P_{old}, is determined by the steady state of the model that describes the pre-label-switch system:

$$\frac{\mathrm{d}}{\mathrm{d}t}\mathrm{P}_{pre}(t) = -\mathrm{P}_{pre}(t)\, k_{deg} + k_{syn} = 0. \tag{5}$$

The initial condition of species P_{new} is 0 because at label switch all protein is old.

The link between a model and the available data is obtained by the definition of model *observables*, which represent the data as a function of the model species. For metabolic labeling experiments, the new protein synthesized after label switch is distinguished from old protein by incorporation of isotopically labelled amino acids. The isotopologue ratio of new and old protein is measured by mass spectrometry, e.g., on the MS1-level [15]. This ratio constitutes the raw data. Accordingly, we can define the observable of Model 0:1 as the fraction of old protein remaining at time t, which is derived as follows:

$$\mathrm{O}(t) = \left(\frac{\mathrm{P}_{new}(t)}{\mathrm{P}_{old}(t)} + 1\right)^{-1} = \frac{\mathrm{P}_{old}(t)}{\mathrm{P}_{new}(t) + \mathrm{P}_{old}(t)} \tag{6}$$

$$= \frac{\mathrm{P}_{old}(t)}{\mathrm{P}_{old+new}} \tag{7}$$

Here the model species ratio $\frac{P_{new}(t)}{P_{old}(t)}$ is the model representation of the raw data and we add 1 and take the reciprocal in order to derive the intuitive interpretation as the fraction of old protein remaining. It can be shown that $\frac{d}{dt}P_{old}(t) = -\frac{d}{dt}P_{new}(t)$. Therefore, the sum of old and new protein is a constant, which we denote as $P_{old+new}$, given by the initial abundance of the old protein:

$$P_{new}(t) + P_{old}(t) = \mathit{const.} = P_{old,0} = \frac{k_{syn}}{k_{deg}} \equiv P_{old+new}, \tag{8}$$

which was used to derive equation (7).

3.2 M0:1 Clearance profile and clearance rate

We define the *clearance profile* $\varphi(t)$ as the natural logarithm of the observable (the fraction of old protein remaining at time t). For Model M0:1 the clearance profile is given by

$$\varphi(t) = \ln\left(O(t)\right) = \ln\left(\frac{P_{old}(t)}{P_{old+new}}\right). \tag{9}$$

$P_{old}(t)$ can be derived from (1) and (3) to be

$$P_{old}(t) = \frac{k_{syn}}{k_{deg}}\, e^{-k_{deg}t}. \tag{10}$$

Substituting (10) and (8) in (9), results in the well-known linear relationship

$$\varphi(t) = \ln\left(\frac{\frac{k_{syn}}{k_{deg}}\, e^{-k_{deg}t}}{\frac{k_{syn}}{k_{deg}}}\right) = -k_{deg}\, t. \tag{11}$$

For Model M0:1, the *clearance rate*, which we define as the negative derivative with respect to time (slope) of the clearance profile φ (a straight line in case of M0:1), then recovers the degradation constant from the clearance profile:

$$-\frac{d}{dt}\varphi = k_{deg}. \tag{12}$$

This recovers the well-known fact that the degradation constant of a protein pool can be quantified from the slope of the line through the data on a logarithmic y-axis scale.

3.3 M0:1 Summary

The clearance profile of Model 0:1 allows to quantify the degradation constant of the protein. Given the profiles of several proteins, it is possible to compare the proteolytic stabilities of those proteins. As shown above, in the case of a protein pool as modelled in Model 0:1, the clearance rate is a constant and identical to the degradation constant k_{deg}. With this, the degradation constant can be quantitatively determined from the slope of the clearance profile. However, the clearance profile as well as the clearance rate are not a function of k_{syn} and therefore Model 0:1 cannot be used to quantify the rate of protein synthesis.

3.4 A single homogeneous protein pool - Correction for cell cycle related increase in protein abundance

Model M0:1 assumes that the cells are not growing during the data collection. However, in growing cells the total protein amount doubles during the cell cycle, to keep the protein concentration constant. Therefore, in growing cells the sum of old and new protein after the label switch is not constant (as in (8)) but increases as a function of time. Therfore, Model M0:1 is not valid for growing cells. To describe the dynamics of a homogeneous protein pool in growing cells and to learn how to correct for this effect of the cell cycle we need a slightly different model, described in the following.

In growing cells the dynamics after label switch are described by the following equations

$$\frac{\mathrm{d}}{\mathrm{d}t}\mathrm{P}_{old}(t) = -\mathrm{P}_{\mathrm{old}}(t)\, k_{deg} \tag{13}$$

$$\mathrm{P}_{old+new}(t) = \mathrm{P}_{old+new}(0)\, 2^{\frac{t}{t_{cc}}}, \tag{14}$$

where P_{old} is equivalent to Model M0:1, $\mathrm{P}(t)$ is the total protein abundance (old + new, here not constant) at time t after label switch and t_{cc} is the cell cycle duration, which is readily measurable in most cell culture experiments, or can be estimated from the data.

Upon label switch, all protein is old, therefore the initial conditions are equal for both model species and given by

$$\mathrm{P}_{old}(0) = \mathrm{P}_{old,0} = \frac{k_{syn}}{k_{deg}} \tag{15}$$

$$\mathrm{P}_{old+new}(0) = \mathrm{P}_0 = \frac{k_{syn}}{k_{deg}}. \tag{16}$$

The same reasoning as in Model M0:1 leads to the fraction of old protein remaining as the model observable

$$\mathrm{O}_{cc}(t) = \frac{\mathrm{P}_{old}(t)}{\mathrm{P}_{old+new}(t)}, \tag{17}$$

where the subscript cc indicates that the inflence of the cell cycle has not been corrected for. With this the degradation profile including cell cycle effects becomes

$$\varphi_{cc}(t) = \ln\left(\frac{\mathrm{P}_{old}(t)}{\mathrm{P}_{old+new}(t)}\right) = \ln\left(\frac{\frac{k_{syn}}{k_{deg}}\, e^{-k_{deg}t}}{\frac{k_{syn}}{k_{deg}}\, 2^{\frac{t}{t_{cc}}}}\right) \tag{18}$$

$$= \ln\left(\frac{e^{-k_{deg}t}}{2^{\frac{t}{t_{cc}}}}\right) \tag{19}$$

$$= \ln\left(e^{-k_{deg}t}\right) - \ln\left(2^{\frac{t}{t_{cc}}}\right) \tag{20}$$

$$= -k_{deg}t - \frac{\ln(2)}{t_{cc}}\, t. \tag{21}$$

Comparison of (21) and (11), leads to the relation

$$\varphi_{cc}(t) = \varphi(t) - \frac{\ln(2)}{t_{cc}}\, t. \tag{22}$$

With this, the corrected clearance profile (11) can be derived from an uncorrected clearance profile (21) via the relationship:

$$\varphi(t) = \varphi_{cc}(t) + \frac{\ln(2)}{t_{cc}}\, t. \tag{23}$$

In the same way, $\frac{\ln(2)}{t_{cc}}\, t$ can be used to correct data collected in growing cells at time t after the label switch.

4 Model 1:2 - synthesis followed by modification

The simplest model for modification of a protein by a PTM consists of two protein species: the unmodified protein P_u and a protein carrying the PTM P_p. Here we assume that a protein is synthesized in an unmodified form and is then reversibly modified in a first order reaction (Figure 1 d). In the following subsections we provide the definition of Model 1:2 (M1:2) by means of its dynamics, initial conditions and observables, derive its clearance profile and its clearance rate and provide further analysis of these quantities.

4.1 M1:2 Model definition

For Model 1:2 the dynamics starting at the time of the label switch ($t = 0$) are given by the ODEs of 4 model species corresponding to two protein species synthesised before (old) and after the label switch (new), respectively:

$$\frac{\mathrm{d}}{\mathrm{d}t}\mathrm{P}_{u,old}(t) = \mathrm{P}_{P,old}(t)\,k_e - \mathrm{P}_{u,old}(t)\,(k_{deg,u} + k_w) \tag{24}$$

$$\frac{\mathrm{d}}{\mathrm{d}t}\mathrm{P}_{P,old}(t) = \mathrm{P}_{u,old}(t)\,k_w - \mathrm{P}_{P,old}(t)\,(k_{deg,P} + k_e) \tag{25}$$

$$\frac{\mathrm{d}}{\mathrm{d}t}\mathrm{P}_{u,new}(t) = \mathrm{P}_{P,new}(t)\,k_e - \mathrm{P}_{u,new}(t)\,(k_{deg,u} + k_w) + k_{syn} \tag{26}$$

$$\frac{\mathrm{d}}{\mathrm{d}t}\mathrm{P}_{P,new}(t) = \mathrm{P}_{u,new}(t)\,k_w - \mathrm{P}_{P,new}(t)\,(k_{deg,P} + k_e) \tag{27}$$

with k_{syn} being the synthesis rate; $k_{deg,u}$ the degradation constant of the unmodified species; $k_{deg,P}$ the degradation constant of the modified species; k_e the rate constant of erasing the PTM; and k_w the rate constant for adding (i.e. writing) the PTM to the protein.

The initial conditions are given as

$$\mathrm{P}_{u,old}(0) = \mathrm{P}_{u,old,0} = \frac{k_{syn}\,(k_{deg,P} + k_e)}{k_{deg,u}\,k_{deg,P} + k_{deg,u}\,k_e + k_{deg,P}\,k_w} \tag{28}$$

$$\mathrm{P}_{P,old}(0) = \mathrm{P}_{P,old,0} = \frac{k_{syn}\,k_w}{k_{deg,u}\,k_{deg,P} + k_{deg,u}\,k_e + k_{deg,P}\,k_w} \tag{29}$$

$$\mathrm{P}_{u,new}(0) = 0 \tag{30}$$

$$\mathrm{P}_{P,new}(0) = 0 \tag{31}$$

Here, the initial conditions for the old species, $\mathrm{P}_{u,old}$ and $\mathrm{P}_{P,old}$, are defined by the steady state of the model that describes the pre-switching system:

$$\frac{\mathrm{d}}{\mathrm{d}t}\mathrm{P}_{u,pre}(t) = \mathrm{P}_{p,pre}(t)\,k_e - \mathrm{P}_{u,pre}(t)\,(k_{deg,u} + k_w) + k_{syn} = 0 \tag{32}$$

$$\frac{\mathrm{d}}{\mathrm{d}t}\mathrm{P}_{p,pre}(t) = \mathrm{P}_{u,pre}(t)\,k_w - \mathrm{P}_{p,pre}(t)\,(k_{deg,P} + k_e) = 0. \tag{33}$$

The initial conditions for the new species are 0 because synthesis of the new species only starts at label switch.

Before we go on to define the model observables, in the next section we use some properties of Model 1:2 to reduce the number of model equations from 4 to 2. This makes numerical as well as symbolical calculations more efficient.

4.2 M1:2 Model reduction

Although not directly obvious from the model equations (24) to (27), it can be shown that the sum of old and new of each protein species x ($x := \{u, P\}$), $\mathrm{P}_{x,old+new}$, is constant and given by the total abundance of each protein species which is determined by the initial conditions of the old protein species:

$$\mathrm{P}_{u,old}(t) + \mathrm{P}_{u,new}(t) = const. = \mathrm{P}_{u,old,0} \equiv \mathrm{P}_{u,old+new} \tag{34}$$

$$\mathrm{P}_{P,old}(t) + \mathrm{P}_{P,new}(t) = const. = \mathrm{P}_{P,old,0} \equiv \mathrm{P}_{P,old+new}. \tag{35}$$

Therefore, the system can be reduced to the following two ODEs:

$$\frac{\mathrm{d}}{\mathrm{d}t}\mathrm{P}_{u,old}(t) = \mathrm{P}_{P,old}(t)\,k_e - \mathrm{P}_{u,old}(t)\,(k_{deg,u} + k_w) \tag{36}$$

$$\frac{\mathrm{d}}{\mathrm{d}t}\mathrm{P}_{P,old}(t) = \mathrm{P}_{u,old}(t)\,k_w - \mathrm{P}_{P,old}(t)\,(k_{deg,P} + k_e) \tag{37}$$

with initial conditions

$$\mathrm{P}_{u,old}(0) = \frac{k_{syn}\,(k_{deg,P} + k_e)}{k_{deg,u}\,k_{deg,P} + k_{deg,u}\,k_e + k_{deg,P}\,k_w} = \mathrm{P}_{u,old+new} \tag{38}$$

$$\mathrm{P}_{P,old}(0) = \frac{k_{syn}\,k_w}{k_{deg,u}\,k_{deg,P} + k_{deg,u}\,k_e + k_{deg,P}\,k_w} = \mathrm{P}_{P,old+new} \tag{39}$$

and the two algebraic equations

$$\mathrm{P}_{u,new}(t) = \mathrm{P}_{u,old+new} - \mathrm{P}_{u,old}(t) \tag{40}$$

$$\mathrm{P}_{P,new}(t) = \mathrm{P}_{P,old+new} - \mathrm{P}_{P,old}(t). \tag{41}$$

The choice of describing the system using only the subsystem of old species is arbitrary. An equivalent representation could be obtained from the ODEs for the new species (then with initial conditions of zero), and consequently expressing the old species as the difference between the constant total and new. We chose here to focus on the old species since it results in a more intuitive interpretation of the observables, as defined next.

The model observables connect the model species to the available data. For most proteins, the measured data consist of the isotopologue ratios of the modified species, P_P, and an estimate of the entire protein pool , P (P_P+P_u). P_P is typically defined by a single proteoform-specific peptide, while an estimate of the total protein is given, for instance, by the median of all measured (unmodified) p eptides of the protein. As most of the peptides in each protein can be expected to be shared between a modified proteoform (e.g. defined by a single measurable PTM-containing peptide) and the unmodified proteoform, the median will essentially give an abundance-weighted mean estimate over the two proteoforms. It should be noted that in reality many more proteoforms might (and indeed are likely to) exist simultaneously for a given protein, but due to the inherent limitation of bottom-up proteomics, we are in most cases limited to comparing single proteoform-specific peptides to the rest of the peptides.
The measured isotopologue ratios correspond to $\frac{\mathrm{P}_{P,new}}{\mathrm{P}_{P,old}}$ and $\frac{\mathrm{P}_{u,new}+\mathrm{P}_{P,new}}{\mathrm{P}_{u,old}+\mathrm{P}_{P,old}}$ in the model. Equivalently to Model 0:1 we choose to use transformed versions of the data to derive the observables as the fraction of old protein species remaining at each time t after label switch. Thus, for Model 1:2, the observables are defined as

$$\mathrm{O}_P = \left(\frac{\mathrm{P}_{P,new}(t)}{\mathrm{P}_{P,old}(t)} + 1\right)^{-1} = \frac{\mathrm{P}_{P,old}(t)}{\mathrm{P}_{P,old}(t) + \mathrm{P}_{P,new}(t)} = \frac{\mathrm{P}_{P,old}(t)}{\mathrm{P}_{P,old+new}} \tag{42}$$

for the PTM proteoform and

$$\mathrm{O} = \left(\frac{\mathrm{P}_{u,new}(t) + \mathrm{P}_{P,new}(t)}{\mathrm{P}_{u,old}(t) + \mathrm{P}_{P,old}(t)} + 1\right)^{-1} = \frac{\mathrm{P}_{u,old}(t) + \mathrm{P}_{P,old}(t)}{\mathrm{P}_{u,old+new} + \mathrm{P}_{P,old+new}}. \tag{43}$$

$$= \frac{\mathrm{P}_{old}(t)}{\mathrm{P}_{old+new}}. \tag{44}$$

for the entire protein, where P_{old} refers to total abundance (sum of PTM and unmodified) of old protein left and the constant overall protein abundance is given as a function of the model parameters as

$$\mathrm{P}_{old+new} = \mathrm{P}_{u,old+new} + \mathrm{P}_{P,old+new} \tag{45}$$

$$= \frac{k_{syn}\,(k_{deg,P} + k_e + k_w)}{k_{deg,u}\,k_{deg,P} + k_{deg,u}\,k_e + k_{deg,P}\,k_w}. \tag{46}$$

From equations (39) and (46) the steady state fraction (occupancy) of the PTM species as a function of the model parameters can be calculated to be

$$\frac{\mathrm{P}_{P,old+new}}{\mathrm{P}_{old+new}} = \frac{k_w}{k_{deg,P} + k_e + k_w}. \tag{47}$$

4.3 M1:2 Analytical solutions for model species time course

The differential equations (36) and (37) can be analytically solved with their initial conditions to yield the time course of the individual species

$$\mathrm{P}_{u,old}(t) = \mathrm{P}_{u,old+new}\left(\frac{\lambda_1\, \mathrm{e}^{\lambda_2 t}\,(\lambda_2 + k_{deg,P} + k_e) - \lambda_2\, \mathrm{e}^{\lambda_1 t}\,(\lambda_1 + k_{deg,P} + k_e)}{(k_{deg,P} + k_e)\,(\lambda_1 - \lambda_2)}\right) \tag{48}$$

$$\mathrm{P}_{P,old}(t) = \mathrm{P}_{P,old+new}\left(\frac{\lambda_1\, \mathrm{e}^{\lambda_2 t} - \lambda_2\, \mathrm{e}^{\lambda_1 t}}{\lambda_1 - \lambda_2}\right), \tag{49}$$

with $\mathrm{P}_{u,old+new}$ and $\mathrm{P}_{P,old+new}$ as defined in (38) and (39) and λ_1 and λ_2 given by

$$\lambda_1 = -\frac{k_{deg,u} + k_{deg,P} + k_e + k_w - \sqrt{\gamma}}{2} \tag{50}$$

$$\lambda_2 = -\frac{k_{deg,u} + k_{deg,P} + k_e + k_w + \sqrt{\gamma}}{2} \tag{51}$$

with

$$\gamma = (k_{deg,u} + k_{deg,P} + k_e + k_w)^2 - 4\,(k_{deg,u}\, k_e + k_{deg,P}\, k_w + k_{deg,u}\, k_{deg,P}). \tag{52}$$

Where λ_1 and λ_2 are the eigenvalues of the system matrix

$$\mathrm{A} = \begin{pmatrix} -k_{deg,u} - k_w & k_e \\ k_w & -k_e - k_{deg,P} \end{pmatrix}, \tag{53}$$

which is derived from the model equations (36) and (37). For Model 1:2 λ_1 and λ_2 can only take negative values and λ_1 will always have a smaller absolute value than λ_2.

The sum of the two individual species solutions then gives the time course of the remaining old protein

$$\mathrm{P}_{old}(t) = \mathrm{P}_{old+new}\left(\frac{\lambda_1\, \mathrm{e}^{\lambda_2 t}\;(\lambda_2 + k_{deg,P} + k_e + k_w)}{(\lambda_1 - \lambda_2)\;(k_{deg,P} + k_e + k_w)} - \frac{\lambda_2\, \mathrm{e}^{\lambda_1 t}\;(\lambda_1 + k_{deg,P} + k_e + k_w)}{(\lambda_1 - \lambda_2)\;(k_{deg,P} + k_e + k_w)}\right). \tag{54}$$

4.4 M1:2 Clearance profile and clearance rate

With the clearance profiles $\varphi(t)$ defined as the natural logarithm of the observables (the fraction of old proteoform remaining at time t), the clearance profiles of Model 2:1 become

$$\varphi_P = \ln(\mathrm{O}_P(t)) = \ln\left(\frac{\mathrm{P}_{P,old}(t)}{\mathrm{P}_{P,old+new}}\right) \tag{55}$$

$$\varphi = \ln(\mathrm{O}(t)) = \ln\left(\frac{\mathrm{P}_{u,old}(t) + \mathrm{P}_{P,old}(t)}{\mathrm{P}_{u,old+new} + \mathrm{P}_{P,old+new}}\right) = \ln\left(\frac{\mathrm{P}_{old}(t)}{\mathrm{P}_{old+new}}\right). \tag{56}$$

for the PTM species and the entire protein. Note that by dividing the time course function of the PTM species by the total abundance of that species, k_{syn} cancels out and the profile equation becomes independent of k_{syn}. The same is true for φ, the profile of the entire protein. Both profiles therefore do not contain any information on k_{syn}, and k_{syn} can never be quantified from the data we consider.

The clearance rates are then given by the negative of the derivative of the profile with respect to time:

$$\frac{\mathrm{d}}{\mathrm{d}t}\varphi_p = -\frac{\mathrm{d}}{\mathrm{d}t}\left(\ln\left(\frac{\mathrm{P}_{P,old}(t)}{\mathrm{P}_{P,old+new}}\right)\right) \tag{57}$$

$$= -\frac{\frac{\mathrm{d}}{\mathrm{d}t}\mathrm{P}_{P,old}(t)}{\mathrm{P}_{P,old}(t)} \tag{58}$$

$$\frac{\mathrm{d}}{\mathrm{d}t}\varphi = -\frac{\mathrm{d}}{\mathrm{d}t}\left(\ln\left(\frac{\mathrm{P}_{u,old}(t) + \mathrm{P}_{P,old}(t)}{\mathrm{P}_{u,old+new} + \mathrm{P}_{P,old+new}}\right)\right) \tag{59}$$

$$= -\frac{\frac{\mathrm{d}}{\mathrm{d}t}\mathrm{P}_{u,old}(t) + \frac{\mathrm{d}}{\mathrm{d}t}\mathrm{P}_{P,old}(t)}{\mathrm{P}_{u,old}(t) + \mathrm{P}_{P,old}(t)} \tag{60}$$

for the PTM species and the entire protein pool, respectively.

4.5 M1:2 the PTM clearance rate is always less or equal to the clearance rate of the entire protein pool

To show that the clearance rate of the PTM species $\frac{\mathrm{d}}{\mathrm{d}t}\varphi_P$ is always less than or equal to the clearance rate of the entire protein pool $\frac{\mathrm{d}}{\mathrm{d}t}\varphi$, irrespective of the parameter values, we need to show that

$$-\frac{\frac{\mathrm{d}}{\mathrm{d}t}\mathrm{P}_{P,old}(t)}{\mathrm{P}_{P,old}(t)} \leq -\frac{\frac{\mathrm{d}}{\mathrm{d}t}\mathrm{P}_{u,old}(t) + \frac{\mathrm{d}}{\mathrm{d}t}\mathrm{P}_{P,old}(t)}{\mathrm{P}_{u,old}(t) + \mathrm{P}_{P,old}(t)}. \tag{61}$$

Via rearrangements this leads to

$$\frac{\mathrm{d}}{\mathrm{d}t}\mathrm{P}_{P,old}(t)\,\mathrm{P}_{u,old}(t) - \mathrm{P}_{P,old}(t)\,\frac{\mathrm{d}}{\mathrm{d}t}\mathrm{P}_{u,old}(t) \geq 0. \tag{62}$$

Replacing the derivatives with the respective model equations ((36) and (37)) and inserting the analytical solutions of the model species ((48) and (49)) results in

$$\frac{{k_{syn}}^2\,k_w\,\mathrm{e}^{-t\,(k_{deg,u}+k_{deg,P}+k_e+k_w)}}{k_{deg,P}\,k_w + k_{deg,u}\,\left(k_{deg,P}+k_e\right)} \geq 0 \tag{63}$$

which must be true since we assume all parameters to be positive.

This proofs that the clearance rate of the PTM species is always less or equal to the clearance rate of the entire protein pool, irrespective of particular parameter values and therefore irrespective of whether $k_{deg,P}$ is smaller or greater than $k_{deg,u}$. Therefore, a comparison between the clearance rates of the PTM species and the entire protein does not contain the information which effect the PTM has on the the protein's stability. This in in contrast to Model 0:1 where the comparison of the clearance rates of two distinct proteins indicates which of the two proteins is more stable.

4.6 M1:2 clearance profile curvature as measured by the y-axis intercept of the linear section of the profile

The clearance profiles of the PTM species and the entire protein pool become linear and parallel at large times after label switch. This indicates that the clearance rates have become equal to the negative of the eigenvalue with the smallest absolute value (see Section 6 for explanation). To quantify the curvature of a clearance profile before it becomes linear, the intercept of the extension of its linear part with the y-axis can be used (see Supplementary Figure 1 for illustration). With curvature we mean the extend of deviation of a clearance profile from linearity, e.g. whether a clearance profile is concave up (clearance rate increases over time) or concave down (clearance rate decreases over time) and to what extent.

The y-axis intercept of the linear part of each clearance profile can be derived via the analytical solutions of the species dynamics ((48) and (49)) (see Section 6 for explanation and MATLAB file M12_red_symbolic_analysis.m for symbolic derivation) which leads to

$$\mathrm{Y}_P = \ln\left(-\frac{\lambda_2}{\lambda_1 - \lambda_2}\right) \tag{64}$$

$$\mathrm{Y} = \ln\left(-\frac{\lambda_2\,\left(\lambda_1 + k_{deg,p} + k_e + k_w\right)}{\lambda_1 - \lambda_2}\right) - \ln\left(k_{deg,P} + k_e + k_w\right) \tag{65}$$

for the intercepts of the linear parts of the PTM and entire protein pool clearance profiles, respectively. Again, λ_1 and λ_2 are as given in (50) and (51). Given that λ_1 and λ_2 are negative with λ_1 having the smaller absolute value, Y_P must always be positive. This is also illustrated in Supplementary Figure 10 for a set of randomly sampled parameter values. In contrast, the sign of Y depends on which model species has the higher degradation constant. If $k_{deg,P} > k_{deg,u}$ (PTM degron) Y is positive or 0, if $k_{deg,P} < k_{deg,u}$ (PTM stabilon) Y is negative or 0 as shown in Supplementary Figure 11 for the same sample of parameters. This corresponds to a concave up shape of φ for parameter sets constituting PTM degrons and a concave down shape of φ for parameter sets constituting PTM stabilons. Notably, many parameter sets in our random sample result in Y having a value close to 0 (66% for -0.05 $\leq \mathrm{Y}_P \leq$ 0.05), irrespective whether the parameter set corresponds to a PTM degron or a PTM stabilon scenario. This cases correspond to close to linear clearance profiles φ.

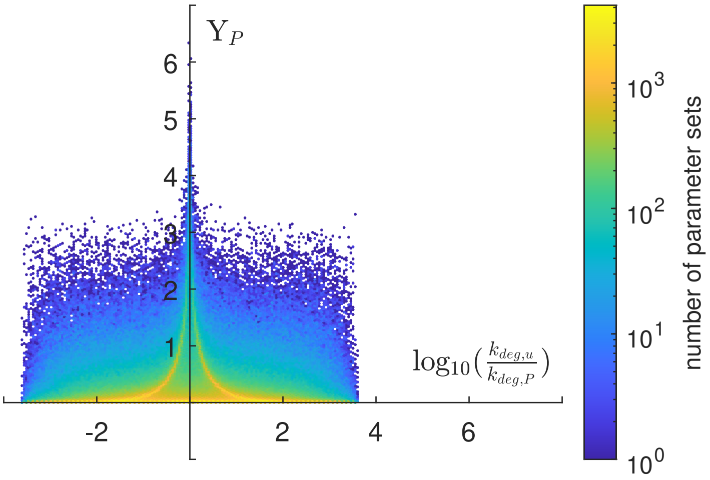

Supplementary Figure 10: **The clearance profile of the PTM species is either linear or concave down.** Value of the y-axis intercept of the linear part of the clearance profile of $P_{P,old}$ (55) versus the logarithm of the ratio between the degradation constants, as calculated for 1 million sets of parameters. Each of the four parameters of Model 1:2 was sampled uniformly on a log2 scale between -12 and 0.

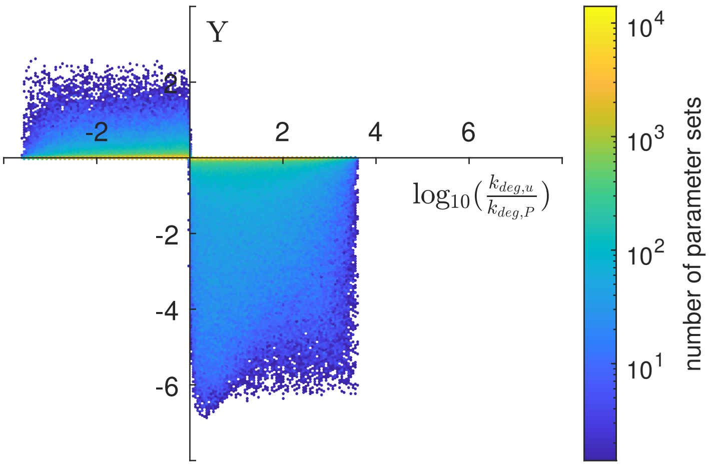

Supplementary Figure 11: **The clearance profile of the entire protein pool is either linear or concave up (PTM degron) or concave down (PTM stabilon).** Value of the y-axis intercept of the linear part of the clearance profile of P_{old} (56) versus the logarithm of the ratio between the degradation constants, as calculated for 1 million sets of parameters. Each of the four parameters of Model 1:2 was sampled uniformly on a log2 scale between -12 and 0.

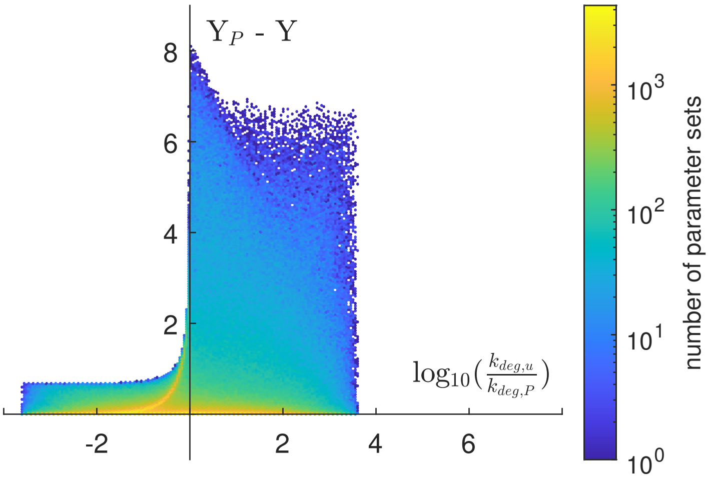

Supplementary Figure 12: **The distance between the profiles of PTM species and entire protein is always positive.** Difference between the y-axis intercepts of the linear parts of the clearance profiles versus the logarithm of the ratio between the degradation constants, as calculated for 1 million random sets of parameters. Each of the four parameters of Model 1:2 was sampled uniformly on a log2 scale between -12 and 0.

The difference between the y-axis intercepts of the linear parts of the clearance profiles of PTM and the entire protein pool is given by

$$Y_P - Y = \ln\left(\frac{k_{deg,P} + k_e + k_w}{\lambda_1 + k_{deg,P} + k_e + k_w}\right), \tag{66}$$

is a quantitative measure of the maximum difference between the clearance rate of PTM and total protein. It must always be positive since λ_1 is negative and therefore the denominator is smaller than the numerator. Supplementary Figure 12 illustrates this for a random sample of parameter values. Supplementary Figure 13 illustrates the relationship between values of the four model parameters and the observed differences between Y$_P$ and Y.

4.7 M1:2 Structural identifiability analysis

In the same way that model M0:1 is frequently used to quantify the degradation constant (or half life) of protein pools, models could be used to estimate the model parameters from experimental clearance profile data. Quantitative values for the parameters could then be used to determine the effect of a PTM on protein stability, i.e. to call PTM degrons and PTM stabilons. The first step in the process of parameter estimation should always be a structural identifiability analysis of the model [16].

Structural identifiability analysis answers the question whether it is possible to quantify the unknown parameters of a model by means of an analysis solely considering the model structure, i.e. the model equations together with the initial conditions and the observables. Since the analysis does not consider any data it is also called *a priori* identifiability analysis. Structural identifiability is a binary model property - a model is either identifiable or not. A model is structurally identifiable if all of the unknown parameters are structurally identifiable. If one or more parameters are not structurally identifiable (structurally unidentifiable) the model is not structurally identifiable. Structural identifiability is a prerequisite for the quantification of a parameter and its uncertainty from real data. However, how well a parameter can be quantified, ultimately depends on the quality and quantity of the data.

There are multiple methods to perform a structural identifiability analysis, some of which have a readily available implementation [17]. The user provides the model structure (model equations including inputs + initial conditions+ observables) and the analysis tool returns whether the provided model structure is structurally identifiable, usually together with information on the identifiability of the individual parameters. We used DAISY [18] and STRIKE-GOLDD [19] to analyse Model 1:2. The analysis revealed that out of the five model parameters only $k_{deg,u}$ is structurally identifiable.

We analysed several variants of Model 1:2 that differ in the model observables. In addition to the observable defined in (42) and (44) we consider the fraction of old unmodified protein remaining as a

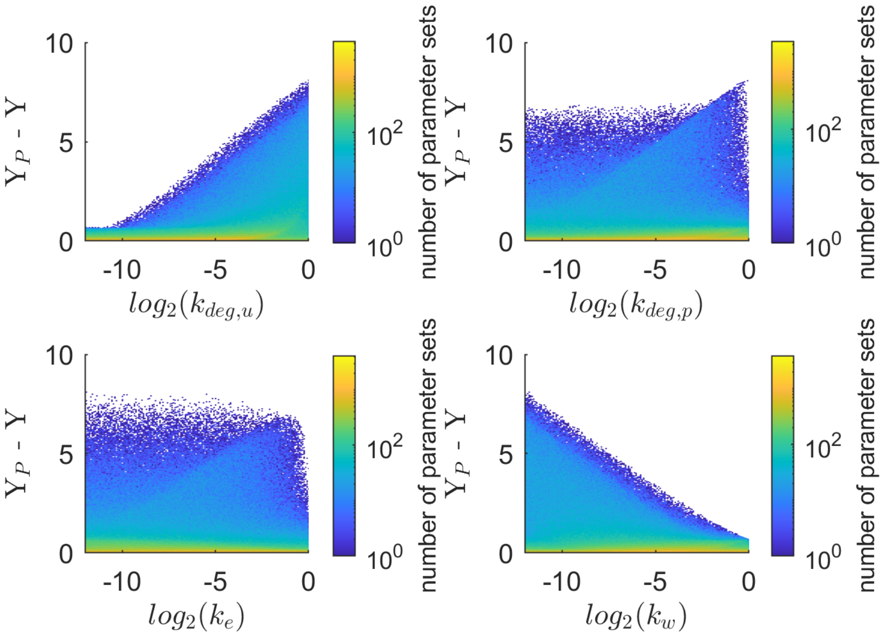

Supplementary Figure 13: **A PTM species profile that is much slower than the profile of the entire protein is caused by a big $k_{deg,u}$ in combination with a small k_w.** Plots show the difference between the y-axis intercepts of the linear parts of the clearance profiles over the log2 of each of the four parameters in each of the 1 million random sets of parameters.

O	O_P	O_u	k_{syn}	$k_{deg,u}$	$k_{deg,P}$	k_e	k_w
x	x			x			
	x	x					
x		x		x	x	x	x

Supplementary Table 1: Observables in different variants of Model 1:2 and structurally identifiable parameters are indicated by "x" in the respective column. The first row corresponds to Model 1:2.

possible observable:

$$O_u = \left(\frac{P_{u,new}(t)}{P_{u,old}(t)} + 1\right)^{-1} = \frac{P_{u,old}(t)}{P_{u,old}(t) + P_{u,new}(t)} = \frac{P_{u,old}(t)}{P_{u,old+new}}. \tag{67}$$

Table 1 summarises the the identifiability properties of the model variants defined by the set of observables. In summary, k_{syn} is unidentifiable for all three theoretically possible observable combinations. This is expected since the observable are not a function of k_{syn}. To make all other parameters identifiable one would need to measure the remaining fraction of the old unmodified species in addition to the remaining fraction of the total old protein. Notably, obtaining the clearance profiles of the two most readily-measurable species (the modified species P_P, which can be enriched with specific PTM-enrichment, and the total P, which can be estimated from all unmodified peptides of the protein) only allows for structural identifiability of $k_{deg,u}$. Furthermore, as the unmodified species P_u is only biologically meaningful in conjunction with its designated modified counterpart, measuring it alone (while being the most information rich for the wiring scheme encoded in Model 1:2 from the standpoint of identifiability) would not readily yield biological insight.

4.8 M1:2 Summary

For Model 1:2 the order of the clearance profiles (slower/faster) contains no information about the proteolytic stability ($k_{deg,x}$) of the different species. The profile of the modified species is always slower then the profile of the entire protein. The clearance rates are functions of time. The clearance rate of the modified species starts at 0 and increases until it becomes constant at λ_1, The clearance rate of the entire

protein starts at a non-zero value and decreases or increases (depending on whether its a PTM stabilon or PTM degron) until it reaches λ_1.

5 Model 1:3 - alternative path to modification via an additional protein species

Adding one more unmodified proteoform to the mechanism, we can derive a general three-species model consisting of two unmodified species and one PTM protein species (Supplementary Figure 8). We assume that a protein is synthesized in an unmodified form, P_{u1}, and then this species is either directly modified as in Model 1:2, or can access another unmodified state P_{u2}. Here, P_{u2} corresponds to a distinct protein pool defined by some other means, such as other PTMs, distinct subcellular localization, protein-protein interaction assemblies, or some other feature, which would make it behave in a distinct manner from the other unmodified proteoform P_{u1}.

Then the model is defined by the following ODEs:

$$\dot{\mathrm{P}}_{u1,old}(t) = \mathrm{P}_{P,old}\, k_{e1} + \mathrm{P}_{u2,old}\, k_{rev} - \mathrm{P}_{u1,old}\,(k_{deg,u1} + k_{w1} + k_{trans}) \quad (68)$$

$$\dot{\mathrm{P}}_{u2,old}(t) = \mathrm{P}_{P,old}\, k_{e1} + \mathrm{P}_{u1,old}\, k_{trans} - \mathrm{P}_{u2,old}\,(k_{deg,u2} + k_{rev} + k_{w2}) \quad (69)$$

$$\dot{\mathrm{P}}_{P,old}(t) = \mathrm{P}_{u1,old}\, k_{w1} + \mathrm{P}_{u2,old}\, k_{w2} - \mathrm{P}_{P,old}\,(k_{deg,P} + k_{e1} + k_{e2}) \quad (70)$$

$$\dot{\mathrm{P}}_{u1,new}(t) = \mathrm{P}_{P,new}\, k_{e1} + \mathrm{P}_{u2,new}\, k_{rev} - \mathrm{P}_{u1,new}\,(k_{deg,u1} + k_{w1} + k_{trans}) + k_{syn} \quad (71)$$

$$\dot{\mathrm{P}}_{u2,new}(t) = \mathrm{P}_{P,new}\, k_{e1} + \mathrm{P}_{u1,new}\, k_{trans} - \mathrm{P}_{u2,new}\,(k_{deg,u2} + k_{rev} + k_{w2}) \quad (72)$$

$$\dot{\mathrm{P}}_{P,new}(t) = \mathrm{P}_{u1,new}\, k_{w1} + \mathrm{P}_{u2,new}\, k_{w2} - \mathrm{P}_{P,new}\,(k_{deg,P} + k_{e1} + k_{e2}) \quad (73)$$

where each of the protein species has its individual degradation constant $k_{deg,x}$ with $x = \{u1,\, u2,\, p\}$, and the remaining rate constants describe the kinetics of interchange between the different protein forms.

The initial conditions are:

$$\mathrm{P}_{u1,old}(0) = \mathrm{P}_{u1,old,0} \quad (74)$$

$$\mathrm{P}_{u2,old}(0) = \mathrm{P}_{u2,old,0} \quad (75)$$

$$\mathrm{P}_{P,old}(0) = \mathrm{P}_{P,old,0} \quad (76)$$

$$\mathrm{P}_{u1,new}(0) = 0 \quad (77)$$

$$\mathrm{P}_{u2,new}(0) = 0 \quad (78)$$

$$\mathrm{P}_{P,new}(0) = 0 \quad (79)$$

The initial conditions for the old species, $\mathrm{P}_{u1,old}$, $\mathrm{P}_{u2,old}$ and $\mathrm{P}_{P,old}$, are defined by the steady state of the model that describes the pre-switching system:

$$\dot{\mathrm{P}}_{u1,pre}(t) = \mathrm{P}_{p,pre}\, k_{e1} + \mathrm{P}_{u2,pre}\, k_{rev} - \mathrm{P}_{u1,pre}\,(k_{deg,u1} + k_{w1} + k_{trans}) = 0 \quad (80)$$

$$\dot{\mathrm{P}}_{u2,pre}(t) = \mathrm{P}_{p}\, k_{e1} + \mathrm{P}_{u1,pre}\, k_{trans} - \mathrm{P}_{u2,pre}\,(k_{deg,u2} + k_{rev} + k_{w2}) = 0 \quad (81)$$

$$\dot{\mathrm{P}}_{p,pre}(t) = \mathrm{P}_{u1}\, k_{w1} + \mathrm{P}_{u2,pre}\, k_{w2} - \mathrm{P}_{p}\,(k_{deg,P} + k_{e1} + k_{e2}) = 0 \quad (82)$$

while the ones for the new species are 0 since all protein is old.

The measured isotopologue ratios correspond to $\frac{\mathrm{P}_{P,new}}{\mathrm{P}_{P,old}}$ and $\frac{\mathrm{P}_{u,new}+\mathrm{P}_{P,new}}{\mathrm{P}_{u,old}+\mathrm{P}_{P,old}}$ in the model. Equivalently to the other models, we choose to use transformed versions of the data to derive the observables as the fraction of old protein species remaining at each time t after label switch. Thus, for Model 1:3, the observables are defined as

$$\mathrm{O}_P = \left(\frac{\mathrm{P}_{P,new}(t)}{\mathrm{P}_{P,old}(t)} + 1\right)^{-1} = \frac{\mathrm{P}_{P,old}(t)}{\mathrm{P}_{P,old}(t) + \mathrm{P}_{P,new}(t)} = \frac{\mathrm{P}_{P,old}(t)}{\mathrm{P}_{P,old+new}} \quad (83)$$

for the PTM proteoform and

$$\mathrm{O} = \left(\frac{\mathrm{P}_{u1,new}(t) + \mathrm{P}_{u2,new}(t) + \mathrm{P}_{P,new}(t)}{\mathrm{P}_{u1,old}(t) + \mathrm{P}_{u2,old}(t) + \mathrm{P}_{P,old}(t)} + 1 \right)^{-1} \tag{84}$$

$$= \frac{\mathrm{P}_{u1,old}(t) + \mathrm{P}_{u2,old}(t) + \mathrm{P}_{P,old}(t)}{\mathrm{P}_{u1,old+new} + \mathrm{P}_{u2,old+new} + \mathrm{P}_{P,old+new}}. \tag{85}$$

$$= \frac{\mathrm{P}_{old}(t)}{\mathrm{P}_{old+new}}. \tag{86}$$

for the entire old protein pool (sum of PTM and unmodified), where P_{old} refers to the abundance of old protein left and $\mathrm{P}_{old+new}$ is the constant abundance of the entire protein pool.

6 General analysis of clearance profiles and clearance rates

We define the *clearance profile* as the natural logarithm of the fraction of old protein of species P_x remaining, $\varphi_x(t)$, at time t, for any protein species P_x as:

$$\varphi_x(t) = \ln \left(\frac{\mathrm{P}_{x,old}(t)}{\mathrm{P}_{x,old+new}} \right) \tag{87}$$

where the x in the subscript is used to refer to the different model species in case there is more than one. Accordingly, we define the *clearance rate* as the negative of the derivative of the clearance profile with respect to time (the slope) as

$$\frac{\mathrm{d}}{\mathrm{d}t}\varphi_x = \frac{\mathrm{d}}{\mathrm{d}t} \left(\ln \left(\frac{\mathrm{P}_{x,old}(t)}{\mathrm{P}_{x,old+new}} \right) \right) \tag{88}$$

$$= \frac{\frac{\mathrm{d}}{\mathrm{d}t}\mathrm{P}_{x,old}(t)}{\mathrm{P}_{x,old}(t)}. \tag{89}$$

We take the negative for the clearance rate to be a positive number.

To assess the clearance rate we thus need to look the analytical solution of the protein species $\mathrm{P}_{x,old}(t)$ and its derivative only. For linear differential equations, as resulting when assuming zeroth and first order reactions only, it is often possible to derive this solution. In its general form it is given by

$$\mathrm{P}_{x,old}(t) = \sum_i C_i \, \mathrm{e}^{\lambda_i t}, \tag{90}$$

where i is the number of protein species involved in the mechanism (i.e. two for Model 1:2), λ_i is the *ith* eigenvalue of the system matrix A, which is derived from the model equations. The eigenvalues are functions of the parameters, including the degradation constants of each species. The coefficients C_i are constants which are functions of the parameters. The derivative of this solution with respect to time can then be obtained to be

$$\frac{\mathrm{d}}{\mathrm{d}t}\mathrm{P}_{x,old}(t) = \sum_i \lambda_i \, C_i \, \mathrm{e}^{\lambda_i t}. \tag{91}$$

Using this general representation of the solution and its derivative, the clearance profile can be written as

$$\varphi_x(t) = \ln \left(\frac{\sum_i C_i \, \mathrm{e}^{\lambda_i t}}{\mathrm{P}_{x,old+new}} \right). \tag{92}$$

Correspondingly, the general representation of the clearance rate is given as

$$\frac{\mathrm{d}}{\mathrm{d}t}\varphi_x = \frac{\sum_i \lambda_i \, C_i \, \mathrm{e}^{\lambda_i t}}{\sum_i C_i \, \mathrm{e}^{\lambda_i t}}. \tag{93}$$

Equation (93) shows that the clearance rate is not only a function of time, but the relation between the clearance rate and the degradation constants also becomes more complicated, the more species are involved in a wiring scheme.

If we order the eigenvalues by increasing absolute value, the exponential that has λ_1 in the exponent will be the exponential that declines to 0 the slowest. Therefore, this exponential function will be the sole exponential left for large times t:

$$\lim_{t\to\infty} \frac{\mathrm{d}}{\mathrm{d}t}\varphi_x = \frac{\lambda_1\, C_1\, \mathrm{e}^{\lambda_1 t}}{C_1\, \mathrm{e}^{\lambda_1 t}} = \lambda_1. \tag{94}$$

Hence, for large times the clearance profile becomes linear with the clearance rate (negative slope) given by λ_1.

Supplementary References

1. Ochoa, D. *et al.* The functional landscape of the human phosphoproteome. *Nature Biotechnology* **38,** 365–373 (2020).
2. Martinez-Val, A. *et al.* Spatial-proteomics reveals phospho-signaling dynamics at subcellular resolution. *Nature Communications* **12,** 7113 (2021).
3. Giurgiu, M. *et al.* CORUM: the comprehensive resource of mammalian protein complexes-2019. *Nucleic acids research* **47,** D559–D563 (2019).
4. Consortium, U. UniProt: the universal protein knowledgebase in 2021. *Nucleic acids research* **49,** D480–D489 (2021).
5. Herr, P. *et al.* Cell Cycle Profiling Reveals Protein Oscillation, Phosphorylation, and Localization Dynamics. *Molecular & cellular proteomics : MCP* **19,** 608–623 (2020).
6. Schwanhäusser, B. *et al.* Global quantification of mammalian gene expression control. *Nature* **473,** 337–342 (2011).
7. Welle, K. A. *et al.* Time-resolved Analysis of Proteome Dynamics by Tandem Mass Tags and Stable Isotope Labeling in Cell Culture (TMT-SILAC) Hyperplexing. *Molecular & cellular proteomics : MCP* **15,** 3551–3563 (2016).
8. MATLAB. *9.9.0.1570001 (R2020b) Update 4* (The MathWorks Inc., Natick, Massachusetts, 2021).
9. Khaled, N. *Latin Hypercube (https://www.mathworks.com/matlabcentral/fileexchange/45793-latin-hypercube)* (MATLAB Central File Exchange. Retrieved October 05, 2021.).
10. Gordon. *hexscatter.m (https://www.mathworks.com/matlabcentral/fileexchange/45639-hexscatter-m)* (MATLAB Central File Exchange. Retrieved October 06, 2021.).
11. Gameiro, P. A., Encheva, V., Dos Santos, M. S., MacRae, J. I. & Ule, J. Metabolic turnover and dynamics of modified ribonucleosides by 13C labeling. *The Journal of biological chemistry* **297,** 101294 (2021).
12. Guan, S., Price, J. C., Ghaemmaghami, S., Prusiner, S. B. & Burlingame, A. L. Compartment modeling for mammalian protein turnover studies by stable isotope metabolic labeling. *Analytical chemistry* **84,** 4014–4021 (2012).
13. Fornasiero, E. F. *et al.* Precisely measured protein lifetimes in the mouse brain reveal differences across tissues and subcellular fractions. *Nature communications* **9,** 4230 (2018).
14. Hammond, D. E. *et al.* Harmonizing Labeling and Analytical Strategies to Obtain Protein Turnover Rates in Intact Adult Animals. *Molecular & cellular proteomics : MCP* **21,** 100252 (2022).
15. Mathieson, T. *et al.* Systematic analysis of protein turnover in primary cells. *Nature communications* **9,** 689 (2018).
16. Villaverde, A. F., Pathirana, D., Fröhlich, F., Hasenauer, J. & Banga, J. R. A protocol for dynamic model calibration. *Briefings in bioinformatics* **23** (2022).
17. Chis, O.-T., Banga, J. R. & Balsa-Canto, E. Structural identifiability of systems biology models: a critical comparison of methods. *PloS one* **6,** e27755 (2011).

18. Bellu, G., Saccomani, M. P., Audoly, S. & D'Angiò, L. DAISY: a new software tool to test global identifiability of biological and physiological systems. *Computer methods and programs in biomedicine* **88,** 52–61 (2007).
19. Villaverde, A. F., Barreiro, A. & Papachristodoulou, A. Structural Identifiability of Dynamic Systems Biology Models. *PLoS computational biology* **12,** e1005153 (2016).
