# Supplementary material for: Protein-Peptide Turnover Profiling reveals the order of PTM addition and removal during protein maturation
Source: Nat Commun. 2022 Dec 2;13:7431. doi: 10.1038/s41467-022-35054-2 (PMC9718778; doi:10.1038/s41467-022-35054-2)
Supplement: Supplementary file 3 — Description of Additional Supplementary Files [file 41467_2022_35054_MOESM3_ESM.docx]

Description of Additional Supplementary Files

File Name: Supplementary_Data_1.xlsx

Description: Protein-level information

File Name: Supplementary_Data_2.xlsx

Description: Peptide-level statistics

File Name: Supplementary_Data_3.xlsx

Description: Raw data from PPToP

File Name: Supplementary_Data_4.xlsx

Description: GFP-fusion constructs used

File Name: Supplementary_Data_5.xlsx

Description: Key resources
