# Supplementary material for: Protein-Peptide Turnover Profiling reveals the order of PTM addition and removal during protein maturation
Source: Nat Commun. 2022 Dec 2;13:7431. doi: 10.1038/s41467-022-35054-2 (PMC9718778; doi:10.1038/s41467-022-35054-2)
Supplement: Supplementary file 9 — Reporting Summary [file 41467_2022_35054_MOESM9_ESM.pdf]

nature portfolio

Corresponding author(s): Henrik Hammarén, Martin Beck, Mikhail Savitski

Last updated by author(s): Nov 11, 2022

Reporting Summary

Nature Portfolio wishes to improve the reproducibility of the work that we publish. This form provides structure for consistency and transparency in reporting. For further information on Nature Portfolio policies, see our Editorial Policies and the Editorial Policy Checklist.

Statistics

For all statistical analyses, confirm that the following items are present in the figure legend, table legend, main text, or Methods section.

n/a	Confirmed	
☐	☒	The exact sample size (*n*) for each experimental group/condition, given as a discrete number and unit of measurement
☐	☒	A statement on whether measurements were taken from distinct samples or whether the same sample was measured repeatedly
☐	☒	The statistical test(s) used AND whether they are one- or two-sided<br>*Only common tests should be described solely by name; describe more complex techniques in the Methods section.*
☐	☒	A description of all covariates tested
☐	☒	A description of any assumptions or corrections, such as tests of normality and adjustment for multiple comparisons
☐	☒	A full description of the statistical parameters including central tendency (e.g. means) or other basic estimates (e.g. regression coefficient) AND variation (e.g. standard deviation) or associated estimates of uncertainty (e.g. confidence intervals)
☐	☒	For null hypothesis testing, the test statistic (e.g. *F*, *t*, *r*) with confidence intervals, effect sizes, degrees of freedom and *P* value noted<br>*Give P values as exact values whenever suitable.*
☐	☒	For Bayesian analysis, information on the choice of priors and Markov chain Monte Carlo settings
☐	☒	For hierarchical and complex designs, identification of the appropriate level for tests and full reporting of outcomes
☐	☒	Estimates of effect sizes (e.g. Cohen's *d*, Pearson's *r*), indicating how they were calculated

Our web collection on statistics for biologists contains articles on many of the points above.

Software and code

Policy information about availability of computer code

Data collection	Proteomic data was collected using Xcalibur (4.3) (Thermo) and analyzed using MaxQuant (version 1.6.4.0) or Isobarquant (Franken et al 2015).
Data analysis	Analysis of the proteomic data was carried out in R (version 4.0.0 or later). Symbolic and numeric computations for the Supplementary Note 1 were performed in MATLAB (9.9.0.1570001 (R2020b) Update 4).

For manuscripts utilizing custom algorithms or software that are central to the research but not yet described in published literature, software must be made available to editors and reviewers. We strongly encourage code deposition in a community repository (e.g. GitHub). See the Nature Portfolio guidelines for submitting code & software for further information.

Data

Policy information about availability of data

All manuscripts must include a data availability statement. This statement should provide the following information, where applicable:

- Accession codes, unique identifiers, or web links for publicly available datasets
- A description of any restrictions on data availability
- For clinical datasets or third party data, please ensure that the statement adheres to our policy

The mass spectrometry proteomics data generated in this study have been deposited in the ProteomeXchange Consortium database via the PRIDE 43 partner repository under the following accession codes
PXD033254 [http://proteomecentral.proteomexchange.org/cgi/GetDataset?ID=PXD033254] (PPToP proteome-wide exploratory dataset)
PXD032945 [http://proteomecentral.proteomexchange.org/cgi/GetDataset?ID=PXD032945] (AP-MS validation dataset of GFP fusion proteins)
The processed data including peptide-level statistics used in this study are provided in the Supplementary Information.
Source data for figures showing quantitative data (46 individual sheets) are provided with this paper.
Reference data used in this study are accessible as follows:
Uniprot database: https://www.uniprot.org/
Annotation of phosphosites from Ref 14: Supplementary information of original publication [https://static-content.springer.com/esm/art%3A10.1038%2Fs41587-019-0344-3/MediaObjects/41587_2019_344_MOESM5_ESM.xlsx]
Phosphoproteomics data along the cell cycle from Ref 22 Supplementary information of original publication [https://www.mcponline.org/cms/10.1074/mcp.RA120.001938/attachment/dc10ab48-f218-42f7-ad01-940cd305a346/mmc1.zip]
Predicted degrons from Ref 25: http://degron.phasep.pro/ [http://degron.phasep.pro/]
Verified degrons from Ref 26: degrons [http://dosztanyi.web.elte.hu/CANCER/DEGRON/TP.html]
Structure of PSMA5 in the proteasome: PDB ID: 6MSB [https://www.ebi.ac.uk/pdbe/entry/pdb/6MSB]
Database of core protein complexes at CORUM [http://mips.helmholtz-muenchen.de/corum/download/releases/old/corum_2018_09_03.zip]
D2P2 database of intrinsically disordered proteins: https://d2p2.pro/ [https://d2p2.pro/]
Occupancy estimates for phosphosites from Ref 32 Supplementary information of original publication [https://pubs.acs.org/doi/suppl/10.1021/acs.jproteome.7b00571/suppl_file/pr7b00571_si_003.xlsx]
Number of studies for each phosphosite: PhosphositePlus [https://www.phosphosite.org/homeAction.action]
Subcellular localizations of proteins: Human Protein Atlas [https://www.proteinatlas.org]
Subcellular fractionation phosphoproteomic data from Ref 31: Supplementary information of original publication [https://static-content.springer.com/esm/art%3A10.1038%2Fs41467-021-27398-y/MediaObjects/41467_2021_27398_MOESM8_ESM.xlsx]

The quantitative models and their behavior in a pSILAC experiment as well as the experimental data presented in this study can be browsed and visualized via an interactive web application at https://apps.embl.de/pptop.

Human research participants

Policy information about studies involving human research participants and Sex and Gender in Research.

Reporting on sex and gender	The study did not include human research participants.
Population characteristics	Not applicable.
Recruitment	Not applicable.
Ethics oversight	Not applicable.

Note that full information on the approval of the study protocol must also be provided in the manuscript.

Field-specific reporting

Please select the one below that is the best fit for your research. If you are not sure, read the appropriate sections before making your selection.

☒ Life sciences ☐ Behavioural & social sciences ☐ Ecological, evolutionary & environmental sciences

For a reference copy of the document with all sections, see nature.com/documents/nr-reporting-summary-flat.pdf

Life sciences study design

All studies must disclose on these points even when the disclosure is negative.

Sample size	Sample sizes and number of replicates were chosen following standards in the field. As per generally-accepted guidelines for protein turnover studies (see, e.g., Claydon et Beynon, 2012 (https://doi.org/10.1074/mcp.O112.022186)), a higher number of time points was favoured over a higher number of replicates per time point. Thus 2-4 biological replicates (8 time points) where used for the initial proteome-wide screen and 2-3 biological replicates (5 time points) for the AP-MS experiments.
Data exclusions	Data from initial 0 and 0.5 h data points from the proteome-wide screen were omitted due to extremely low or nonexistent SILAC label incorporation at low times. Likewise data from the 28 h timepoint was omitted from the proteome-wide screen due to the large temporal difference to the other datapoints in the time series. Data from the last time point (24 h) in the AP-MS experiments were omitted for proteins with rapid turnover as extremely low TMT signal reporter values suffer from ratio compression.
Replication	Experiments for the proteome-wide screen were performed thrice on different times with different cell culture batches (Replicates 1 and 2 were done from the same cell culture batch). Replicate 4 of the proteome-wide screen was performed using a label switch (HEAVY-to-LIGHT instead of LIGHT-to-HEAVY) to rule out any artefacts caused by the SILAC label. No replicates were selectively excluded. Reproducibility across replicates was filtered for on a peptide-level as described in Methods. For the follow-up experiments, only results robustly reproducing in all replicates were reported (e.g. PSMA5 pull-downs reproduced in 1-3 replicates on three individual occasions).
Randomization	All MS samples were analyzed on shared-use instruments with samples from other users and other projects in between, thus essentially generating a randomized instrument background. AP-MS experiments were also performed as quasi-randomized batches to reduce the probability of batch effects occurring. Data analysis was done using quantitative analysis codes, which treated all sample groups identically, thus preventing potential analyser bias. Physical sample handling for phosphoenriched and unenriched samples were different precluding the possibility of blinding. Thus no explicit blinding was performed.
Blinding	No blinding was performed during data analysis, as the study did not include treatment/control type experiments.

Reporting for specific materials, systems and methods

We require information from authors about some types of materials, experimental systems and methods used in many studies. Here, indicate whether each material, system or method listed is relevant to your study. If you are not sure if a list item applies to your research, read the appropriate section before selecting a response.

Materials & experimental systems

n/a	Involved in the study	
☐	☒	Antibodies
☐	☒	Eukaryotic cell lines
☒	☐	Palaeontology and archaeology
☒	☐	Animals and other organisms
☒	☐	Clinical data
☒	☐	Dual use research of concern

Methods

n/a	Involved in the study	
☒	☐	ChIP-seq
☒	☐	Flow cytometry
☒	☐	MRI-based neuroimaging

Antibodies

Antibodies used	anti-GFP (abcam, ab1218), anti-mouse Alexa 488 (Thermo Fisher Scientific A11001)
Validation	https://www.abcam.com/gfp-antibody-9f9f9-ab1218.html<br>https://www.thermofisher.com/antibody/product/Goat-anti-Mouse-IgG-H-L-Cross-Adsorbed-Secondary-Antibody-Polyclonal/A-11001

Eukaryotic cell lines

Policy information about cell lines and Sex and Gender in Research

Cell line source(s)	HeLa Kyoto, from: S. Narumiya, RRID: CVCL_1922 (https://www.cellosaurus.org/CVCL_1922)<br>HeLa, from: ATCC, RRID:CVCL_0030
Authentication	Cell lines were authenticated when acquired using short tandem repeat analysis.
Mycoplasma contamination	Both cell lines used were verified to be mycoplasma free.
Commonly misidentified lines<br>(See ICLAC register)	Only HeLa cells were used to ensure compatibility with a large body of previously published data. No commonly misidentified cell lines were used in the study.
